# COVID-19 Vaccine Rollouts and the Reproduction of Urban Spatial Inequality: Disparities Within Large US Cities in March and April 2021 by Racial/Ethnic and Socioeconomic Composition

**DOI:** 10.1007/s11524-021-00589-0

**Published:** 2022-02-03

**Authors:** Nicholas V. DiRago, Meiying Li, Thalia Tom, Will Schupmann, Yvonne Carrillo, Colleen M. Carey, S. Michael Gaddis

**Affiliations:** 1 Department of Sociology, University of California, Los Angeles (UCLA), Box 951551, 264 Haines Hall, Los Angeles, CA 90095-1551 USA; 2 California Center for Population Research, University of California, Los Angeles (UCLA), Box 957236, 4284 Public Affairs Building, Los Angeles, CA 90095-7236 USA; 3 Department of Sociology, University of Southern California, 851 Downey Way, Hazel & Stanley Hall 314, Los Angeles, CA 90089-1059 USA; 4 Department of Economics, Cornell University, 109 Tower Road, 404 Uris Hall, Ithaca, NY 14853-2501 USA

**Keywords:** COVID-19, Disparities, Inequality, Neighborhood, Pandemic, Race, Socioeconomic, Spatial, Urban, Vaccine

## Abstract

Rollouts of COVID-19 vaccines in the USA were opportunities to redress disparities that surfaced during the pandemic. Initial eligibility criteria, however, neglected geographic, racial/ethnic, and socioeconomic considerations. Marginalized populations may have faced barriers to then-scarce vaccines, reinforcing disparities. Inequalities may have subsided as eligibility expanded. Using spatial modeling, we investigate how strongly local vaccination levels were associated with socioeconomic and racial/ethnic composition as authorities first extended vaccine eligibility to all adults. We harmonize administrative, demographic, and geospatial data across postal codes in eight large US cities over 3 weeks in Spring 2021. We find that, although vaccines were free regardless of health insurance coverage, local vaccination levels in March and April were negatively associated with poverty, enrollment in means-tested public health insurance (e.g., Medicaid), and the uninsured population. By April, vaccination levels in Black and Hispanic communities were only beginning to reach those of Asian and White communities in March. Increases in vaccination were smaller in socioeconomically disadvantaged Black and Hispanic communities than in more affluent, Asian, and White communities. Our findings suggest vaccine rollouts contributed to cumulative disadvantage. Populations that were left most vulnerable to COVID-19 benefited least from early expansions in vaccine availability in large US cities.

**Supplementary Information:**

The online version contains supplementary material available at 10.1007/s11524-021-00589-0.

## Introduction

### Background: COVID-19 Vaccine Rollouts in the USA

In early 2021, state and local authorities in the USA vaccinated millions of people weekly against coronavirus disease 2019 (COVID-19).[1] Vaccination curbed viral infection and transmission and reduced illness, hospitalization, and death from COVID-19.[2, 3, 4, 5, 6, 7, 8] Vaccines were free countrywide regardless of health insurance coverage. Eligibility progressed in stages per state and local policy. Health care employees received first priority, followed by seniors, workers in designated occupations, and individuals with particular medical conditions.[9]

The most significant expansion in eligibility occurred from late March through April 2021. Doses remained scarce, but most jurisdictions allowed everyone age 16 and older to be vaccinated.[10, 11, 12, 13, 14, 15] On January 1, 2021, 5.5 million people had received at least one dose of a COVID-19 vaccine. That number increased to 30.3 million by February 1, 57.0 million by March 1, 111.1 million by April 1, and 153.7 million by May 1.[16] Growth plateaued in May. Over 206.6 million US residents were at least partially vaccinated by September 1; nearly half of them received their first dose in March or April. Eligibility expansion enabled rapid increases during these months.

Vaccine eligibility rules did not account for two key predictors of the burden of the COVID-19 pandemic in the USA: race/ethnicity and socioeconomic status (SES). At the community level, infection and mortality were more common where low-SES individuals and people of color (POC) comprised more of the population.[17, 18, 19, 20, 21, 22, 23, 9] At the individual level, Black and Hispanic people were disproportionately likely to experience infection, hospitalization, and death.[24, 25, 26, 27, 28, 29, 30, 31, 32, 33, 34] Socioeconomic variables partially mediated racial/ethnic disparities.

Decision-makers might have opted against conditioning vaccine eligibility on racial/ethnic or socioeconomic factors to avoid legal challenges.[35,36] Still, there were viable ways to use vaccine eligibility policy to mitigate inequality.[37] For example, the Advisory Committee on Immunization Practices (ACIP) initially recommended prioritizing essential workers, which would have increased eligibility for low-SES people and POC.[38] Authorities ultimately hewed closer to ACIP’s final recommendations, giving greater weight to advanced age.[39]

Geographic allocation may have been the most promising indirect means of addressing disparities through the rollout of COVID-19 vaccines. SES, race/ethnicity, and geography are tightly linked in the reproduction of inequality in the USA; socioeconomic and racial/ethnic inequalities manifest in space, usually at hyperlocal scales.[40, 41, 42, 43, 44, 45, 46, 47, 48, 49, 50, 51] Prioritizing local geographies in which residents had the highest risks of hardship from COVID-19 probably would have reduced mortality more than the age-based rollouts authorities chose.[52] Regardless of whether demographic targeting was constitutional, spatial targeting could have advanced vaccine equity.

### Motivation: Early Vaccine Distribution and Cumulative Disadvantage

Authorities relied on individual initiative to distribute vaccines outside the health care workforce. This approach favored individuals with internet access, reliable transportation, and flexible schedules. States and localities used first-come, first-served online scheduling for scarce appointments at small numbers of sites. People with reliable internet access and white-collar jobs were better positioned to sign up. Limited locations and timed appointments were disadvantageous for people with restricted transit options and strict or uncertain work schedules, including the poor and many people with disabilities. Barriers to vaccination in March and April 2021 may have reinforced socioeconomic and racial/ethnic disparities.

Concern over vaccine hesitancy in the USA has abounded, but framing vaccination solely as a matter of individual choice obscures structural and material impediments. Researchers mostly attribute stagnant US vaccination rates to misinformation, mistrust in institutions, and political party affiliation [53, 54, 55, 56, 57, 58, 59, 60, 61,62, 63, 64]. At the same time, survey evidence suggests vaccination was linked to SES in Spring 2021[65]. Unvaccinated respondents reported three major economic concerns: taking time off work to get the vaccine, missing work due to side effects, and out-of-pocket costs. Plausible economic determinants of vaccine uptake as eligibility first expanded suggest racial/ethnic and socioeconomic disparities may have arisen.

If disparities persisted through April 2021, vaccine rollouts contributed to cumulative disadvantage [66, 67]. Advantages secure future advantages; inequality begets inequality—including at the neighborhood level.[43, 68, 69, 70, 71, 72, 73, 74, 75, 76, 77, 78] Vaccine rollouts may have propelled a circular process. POC and low-SES communities were most likely to experience serious illness or die from COVID-19. Equitable vaccine distribution would have mitigated racial/ethnic and socioeconomic gaps, but early vaccine distribution did not account for these inequalities. As a result, geographic clusters of unvaccinated people could have emerged, restarting the cycle by facilitating viral transmission [79, 80, 81, 82, 83, 84, 85]. Understanding racial/ethnic and socioeconomic vaccination disparities at the local level identifies harms that marginalized people experienced during the pandemic and helps explain the reproduction of urban spatial inequality in the USA [86, 87, 88, 89].

### Overview

Our analysis provides a unique perspective on socioeconomic, racial/ethnic, and spatial disparities during the pandemic in the USA. Numerous studies have focused on geographic dimensions of COVID-19–related inequality,[90, 91, 92, 93, 94, 95, 96, 97] but few have examined spatial differences in vaccination below the state level.[59, 98, 99, 100] The temporal persistence of geographical vaccination disparities is particularly underexplored. We also contribute a novel dataset[101] that harmonizes initially incompatible sources. And unlike many studies of COVID-19 disparities—even analyses with a geographical focus—we modeled spatial dynamics.

We tested two hypotheses. First, we hypothesized that local areas in which POC and low-SES individuals comprised more of the population had lower vaccination levels in March and April 2021. Second, we hypothesized that, despite lower starting points, the same areas had smaller increases in vaccination between March and April.

We used spatial quantitative methods to test these hypotheses. We estimated associations between vaccination levels and racial/ethnic and socioeconomic composition, adjusting for populations with early eligibility due to age or employment. We collected administrative data on vaccination by postal code, covering eight of the 10 most populous US cities in March and April 2021. We combined these data with demographic estimates and geospatial data from the US Census Bureau. We used spatial interpolation to reconcile reporting irregularities.

We found that, although vaccines were free regardless of health insurance coverage, local vaccination levels in March and April were negatively associated with poverty, enrollment in means-tested public health insurance (e.g., Medicaid), and the uninsured population. By April, vaccination levels in Black and Hispanic communities were only beginning to reach those of Asian and White communities in March. Increases in vaccination were smaller in socioeconomically disadvantaged Black and Hispanic communities than in more affluent, Asian, and White communities. Our findings suggest vaccine rollouts contributed to cumulative disadvantage.

## Data and Methods

### Data

From online public databases maintained by state and local public health authorities, we gathered official counts of individuals with at least one dose of a COVID-19 vaccine in March and April 2021. Only geographically aggregated data were publicly available. We secured them for eight of the 10 most populous US cities: New York, Chicago, Houston, Phoenix, Philadelphia, San Antonio, San Diego, and Dallas (in descending order of population). The vaccination data capture a 3-week window during which eligibility expanded significantly. The number of individuals with at least one dose of a COVID-19 vaccine in the eight cities increased 34.7% from 4.6 to 7.1 million during this period. We present key details of the vaccination data in Table 1; we elaborate in Section e2.1 of the online supplement.

**Table 1:** Vaccination data sources and coverage

City	Source	Time 1	Time 2
New York	New York City Department of Health and Mental Hygiene	March 22, 2021	April 13, 2021
Chicago	Chicago Department of Public Health	March 22, 2021	April 13, 2021
Houston	Texas Department of State Health Services	March 22, 2021	April 11, 2021
Phoenix	Arizona Department of Health Services	March 22, 2021	April 13, 2021
Philadelphia	Philadelphia Department of Public Health	March 21, 2021	April 12, 2021
San Antonio	Texas Department of State Health Services	March 22, 2021	April 11, 2021
San Diego	County of San Diego Health and Human Services Agency	March 21, 2021	April 12, 2021
Dallas	Texas Department of State Health Services	March 22, 2021	April 11, 2021

We used two datasets from the US Census Bureau. We collected demographic data from the 2015–2019 American Community Survey (ACS) Five-Year Estimates[102] and geospatial vector data from the 2019 TIGER/Line Shapefiles.[103] We provide further detail on these sources in Sections e2.2 and e2.3 of the online supplement.

### Unit of Analysis

For brevity and interpretability, we refer to our units of analysis as ZIP Codes, the name for postal codes in the USA. The units of analysis were based on ZIP Codes, but reporting irregularities made ZIP Codes themselves inviable. Where necessary, we used overlay interpolation[104, 105] to exclude populations residing outside city limits. We provide extensive detail on the units of analysis and interpolation in Section e3 of the online supplement.

### Independent Variables

#### Vaccination Priority Populations

We accounted for vaccination priority regulations by adjusting for populations of health care workers and seniors. Specific estimates were unavailable for health care workers, but ACS provided estimated counts of individuals employed in “health care and social assistance.” We also adjusted for the share of the population age 65 or older. These variables were the best available measures of the first groups prioritized for vaccination. We include more information on these variables in Section e2.2 of the online supplement.

#### Socioeconomic Composition

To examine the dependent variable’s association with socioeconomic composition, we included four indicators of SES. Two independent variables estimated health insurance status. Health insurance coverage was not universal in the USA as of the COVID-19 pandemic, and medical care remained expensive and stratified compared to other rich countries.[106, 107] We included variables estimating the share of the population enrolled in Medicaid or other means-tested public health insurance and the share without health insurance altogether. Together, these variables captured populations that were among the least integrated into the US health care system. We also included variables estimating the shares of the population under the federal poverty line and without internet access. We included the latter because making appointments online was usually the best way to secure a vaccine in early 2021. We include more information on our socioeconomic variables in Section e2.2 of the online supplement.

#### Racial/Ethnic Composition

We accounted for racial/ethnic composition because racism causes health inequity in the USA.[108, 109, 110, 111, 112, 113, 114, 115] Although race/ethnicity itself cannot cause anything, distributive systems that allocate resources according to racial/ethnic hierarchies create disparities among racial/ethnic groups.[116, 117, 118, 119, 120] These disparities often surface the net of SES. Including measures of racial/ethnic composition in our models enabled us to examine its direct association with vaccination, adjusting for SES.

Racism, however, is more than a conditional association between an outcome and racial/ethnic composition.[121, 122, 123, 124, 125, 126] It undergirds the gamut of US social, economic, and political processes. The distributions of socioeconomic covariates and unobserved mechanisms were racialized. We analyzed racism in the aggregate by considering direct and indirect pathways—mainly through simulations, described below and in Section e4.3 of the online supplement.

From ACS racial/ethnic categories, we created variables measuring the estimated populations of four mutually exclusive, non-exhaustive racial/ethnic groups: Asian, Black, Hispanic, and White. We defined Hispanic as Hispanic, Latino, or Spanish origin, of any race(s). We defined Black, Asian, and White as non-Hispanic and Black or African American alone, Asian alone, and White alone, respectively. This approach implies a fifth category comprised of non-Hispanic individuals of multiple races or any other race alone. The racial/ethnic variables did not sum to one (100%) unless the estimated population of the fifth category was zero.

We include more information on our framework for race/ethnicity and racial/ethnic variables in Section e2.2 of the online supplement.

### Dependent Variable

The dependent variable approximated the share of each ZIP Code’s vaccine-eligible population that was partially or fully vaccinated against COVID-19. We calculated it by dividing the estimated number of residents with at least one dose of an approved vaccine by the estimated population age 15 and older. This denominator was the best available measure of the population to whom agencies were authorized to administer vaccines in March and April 2021. More information on the dependent variable is available in Section e2 of the online supplement.

### Spatial-Statistical Analysis

We estimated population-weighted regressions with conventional adjustments for spatial clustering [127]. We report spatial error models (SEMs) estimated by maximum likelihood [127, 128, 129, 130, 131, 132]. Standard linear models (SLMs) are ill suited to estimate associations that vary across space. In this analysis, spatial heterogeneity could have arisen from unmeasurable factors such as COVID-19 exposure, hyperlocal idiosyncrasies in the effects or implementation of vaccination policies, and cultural influences. Standard tests[133, 134] strongly suggested SLMs exhibited spatial heterogeneity in our setting. We estimated SEMs with row-standardized $k$ nearest-neighbor weights ($k=8$) [135, 136, 137]. As the Moran’s $I$ test statistics in Table 3 [138] demonstrate, the SEMs eliminated the residual spatial clustering that emerged in the SLMs. The models incorporated city fixed effects to adjust for unmeasured variables that were constant among ZIP Codes within each city,[139] including elements of vaccination policies. Because multiple cities were in Texas, we calculated heteroskedasticity-robust standard errors clustered by state [140].

To illustrate the estimated associations, we simulated outcomes at representative values in the racial/ethnic and socioeconomic distributions of the sample. This approach resembled a marginal effects analysis but accounted for spatial clustering and yielded an overall average rather than a unit-level estimate.[141, 142, 143, 144] We present eight simulated scenarios: ZIP Codes with high Black populations and (1) low SES or (2) high SES; high Hispanic populations and (3) low SES or (4) high SES; high Asian populations and (5) low SES or (6) high SES; and high White populations and (7) low SES or (8) high SES. We defined low and high levels as below the 10th and above the 90th within-city percentiles, respectively.

We provide additional details on all aspects of our analytical approach, including the models and simulations, in Section e4 of the online supplement.

## Results

### Descriptive Findings

**Fig. 1:**
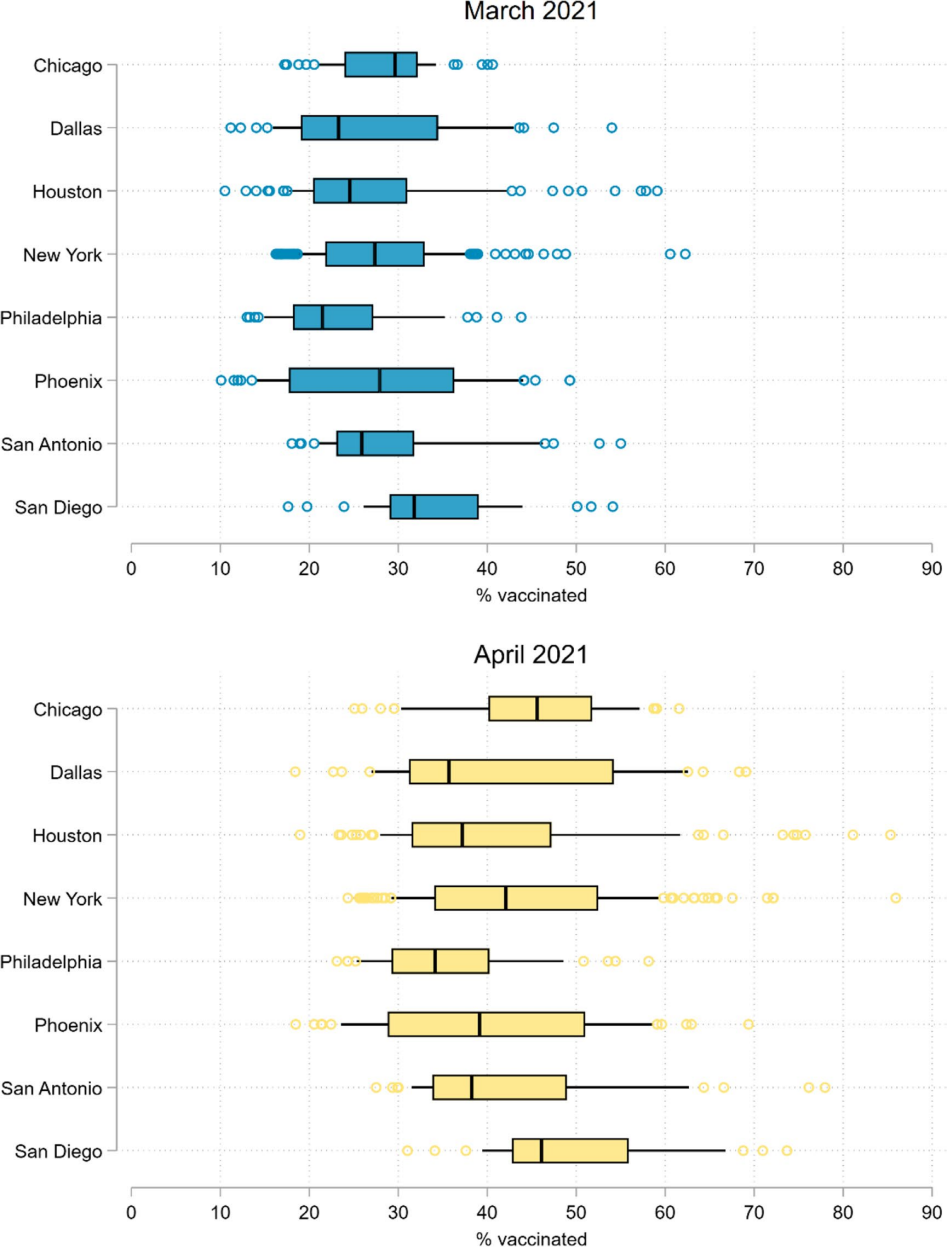
COVID-19 vaccination levels in the population age 15 and older of ZIP Codes in eight large US cities, March and April 2021. Note: Figures are box-and-whisker plots of vaccination levels in $N=552$ ZIP Codes across eight of the 10 most populous US cities. The boxes represent interquartile ranges. The vertical lines represent medians. The horizontal lines extend from the 10th to the 90th percentiles. Circles represent observations below the 10th and above the 90th percentiles. The “% vaccinated” is the percent of the population age 15 and older with at least one dose of a COVID-19 vaccine

In Table 2, we present descriptive statistics at the ZIP Code level. On average across all 552 ZIP Codes, 28.0% of the population in March and 42.4% of the population in April had at least one dose of a COVID-19 vaccine, with a mean difference of 14.5 percentage points (p.p.) between March and April. Other than Philadelphia and San Diego, each city’s mean vaccination level fell within a two-point range (27–29%) in March and a five-point range (40–45%) in April. Although there was some variation between cities, vaccination levels varied considerably more across ZIP Codes within cities (see Fig. 1). In March, the standard deviation in vaccination levels was 3.0 p.p. between cities and 8.8 p.p. within cities; in April, it was 4.2 p.p. between cities and 11.9 p.p. within cities. The mean difference between the 10th and 90th percentiles of vaccination levels across cities was 21.6 p.p. in March and 31.0 p.p. in April.

**Table 2:** Descriptive statistics on COVID-19 vaccination and population composition in ZIP Codes within and across eight large US cities, March and April 2021

	$M$	${SD}$		$M$	${SD}$		$M$	${SD}$
New York ($n=175$)			Chicago ($n=53$)			Houston ($n=99$)		
% vaccinated, March	28.18	7.94	% vaccinated, March	28.70	5.50	% vaccinated, March	27.23	10.03
% vaccinated, April	43.60	11.71	% vaccinated, April	45.08	9.46	% vaccinated, April	40.97	13.64
% vaccinated, difference	15.42	5.24	% vaccinated, difference	16.38	5.21	% vaccinated, difference	13.75	4.03
% 65 +	14.98	5.09	% 65 +	12.55	4.06	% 65 +	10.32	3.09
% health care workers	17.42	6.49	% health care workers	13.93	3.73	% health care workers	10.81	3.31
% under poverty line	15.91	9.42	% under poverty line	17.93	9.90	% under poverty line	18.33	9.36
% w/ Medicaid, etc	16.61	9.96	% w/ Medicaid, etc	12.98	9.48	% w/ Medicaid, etc	6.35	4.13
% w/o health insurance	8.16	4.40	% w/o health insurance	10.13	5.97	% w/o health insurance	25.09	12.31
% w/o internet access	14.62	6.47	% w/o internet access	16.34	9.10	% w/o internet access	16.51	10.85
% Black	19.82	23.39	% Black	29.67	33.53	% Black	22.73	18.63
% Hispanic	26.37	19.34	% Hispanic	22.43	21.94	% Hispanic	42.58	22.27
% Asian	14.77	13.96	% Asian	7.87	8.61	% Asian	6.56	6.23
Phoenix ($n=50$)			Philadelphia ($n=46$)			San Antonio ($n=48$)		
% vaccinated, March	27.78	11.00	% vaccinated, March	23.30	7.65	% vaccinated, March	29.10	9.04
% vaccinated, April	40.17	13.48	% vaccinated, April	35.58	8.74	% vaccinated, April	42.21	11.90
% vaccinated, difference	12.38	3.19	% vaccinated, difference	12.28	2.01	% vaccinated, difference	13.11	3.33
% 65 +	11.32	4.35	% 65 +	14.18	4.61	% 65 +	11.90	3.24
% health care workers	12.08	2.16	% health care workers	20.63	4.38	% health care workers	14.06	2.31
% under poverty line	16.82	10.04	% under poverty line	22.25	11.17	% under poverty line	16.55	8.85
% w/ Medicaid, etc	12.38	7.14	% w/ Medicaid, etc	15.52	9.37	% w/ Medicaid, etc	4.99	3.01
% w/o health insurance	14.37	8.32	% w/o health insurance	8.74	3.58	% w/o health insurance	18.89	8.42
% w/o internet access	13.49	9.36	% w/o internet access	18.16	8.49	% w/o internet access	15.69	9.88
% Black	6.04	4.29	% Black	38.51	30.97	% Black	7.13	7.67
% Hispanic	37.30	23.95	% Hispanic	11.99	13.48	% Hispanic	61.79	20.90
% Asian	3.96	2.77	% Asian	6.96	5.82	% Asian	2.85	2.72
San Diego ($n=33$)			Dallas ($n=48$)			Overall ($N=552$)		
% vaccinated, March	34.16	8.45	% vaccinated, March	27.02	10.40	% vaccinated, March	27.95	9.00
% vaccinated, April	50.30	10.81	% vaccinated, April	42.04	13.92	% vaccinated, April	42.44	12.34
% vaccinated, difference	16.13	3.45	% vaccinated, difference	15.02	4.72	% vaccinated, difference	14.48	4.56
% 65 +	13.18	4.36	% 65 +	10.55	5.72	% 65 +	12.75	4.81
% health care workers	12.87	2.00	% health care workers	11.22	2.42	% health care workers	14.58	5.45
% under poverty line	11.98	6.68	% under poverty line	17.11	9.02	% under poverty line	17.08	9.64
% w/ Medicaid, etc	9.63	7.17	% w/ Medicaid, etc	5.11	3.89	% w/ Medicaid, etc	11.52	9.04
% w/o health insurance	8.11	5.43	% w/o health insurance	24.26	11.89	% w/o health insurance	14.33	10.65
% w/o internet access	7.03	5.30	% w/o internet access	18.29	12.72	% w/o internet access	15.28	9.26
% Black	5.34	4.40	% Black	22.88	19.50	% Black	19.89	23.32
% Hispanic	26.84	21.38	% Hispanic	36.52	20.75	% Hispanic	32.68	23.95
% Asian	15.39	11.40	% Asian	5.23	9.70	% Asian	9.18	10.79

$N=552$ ZIP Codes across eight of the 10 most populous US cities. “Health care workers” refers to individuals employed in health care and social assistance. “Medicaid, etc.” refers to Medicaid or any other means-tested public health insurance. The “% vaccinated” is the percent of the population age 15 and older with at least one dose of a COVID-19 vaccine.

### Model Estimates

In Table 3, we summarize the results of the SEMs with all independent variables for three outcomes: March vaccination levels, April vaccination levels, and the difference between March and April vaccination levels. In both March and April, four variables were significantly associated with the dependent variable. The first, the percent of the population age 65 and older, reflects the policy choice to place older individuals among the earliest priority groups. The other three variables were measures of socioeconomic composition: the shares of the population under the poverty line, with means-tested public health insurance, and without health insurance. Adjusting for vaccination priority populations and racial/ethnic composition, markers of low SES were negatively associated with vaccination levels. In April, vaccination levels were positively associated with the Asian share of the population.

**Table 3:** Spatial error model (SEM) estimates of COVID-19 vaccination levels in the population age 15 and older of ZIP Codes across eight large US cities, March and April 2021

	(1)	(2)	(3)
	% vaccinated, March	% vaccinated, April	Difference
Vaccination priority populations			
% 65 +	0.593***	0.470***	− 0.122*
	(0.048)	(0.075)	(0.054)
% health care workers	0.147	− 0.063	− 0.201***
	(0.257)	(0.309)	(0.055)
Socioeconomic composition			
% under poverty line	− 0.102*	− 0.138**	− 0.039
	(0.051)	(0.051)	(0.023)
% w/ Medicaid, etc	− 0.102***	− 0.127**	− 0.021
	(0.024)	(0.046)	(0.029)
% w/o health insurance	− 0.418***	− 0.655***	− 0.234***
	(0.039)	(0.053)	(0.023)
% w/o internet access	− 0.040	− 0.036	0.003
	(0.051)	(0.060)	(0.011)
Racial/ethnic composition			
% Black	− 0.111	− 0.132	− 0.021
	(0.061)	(0.084)	(0.025)
% Hispanic	0.041	0.076	0.036***
	(0.035)	(0.041)	(0.010)
% Asian	0.101	0.230*	0.127***
	(0.067)	(0.103)	(0.037)
Residual Moran’s$I$			
Standard linear model (SLM)	0.250***	0.222***	0.202***
Spatial error model (SEM)	0.027	0.014	− 0.015

SEMs estimated by maximum likelihood with row-standardized nearest-neighbor spatial weighting ($$k=8$$).$$N=552$$ZIP Codes across eight of the 10 most populous US cities. City fixed effects (reference: New York) and constant terms not shown. Percentages scaled from zero to one. All models weighted by estimated population age 15 and older. Heteroskedasticity-robust standard errors clustered by state in parentheses. ***$$p<0.001$$; **$$p<0.01$$; *$$p<0.05$$. Moran’s$$I$$$$p$$-values calculated by permutation bootstrap (9999 iterations). “Health care workers” refers to individuals employed in health care and social assistance. “Medicaid, etc.” refers to Medicaid or any other means-tested public health insurance. The “% vaccinated” is the percent of the population age 15 and older with at least one dose of a COVID-19 vaccine.

Five variables were significantly associated with differences in vaccination between March and April. The shares of the population age 65 and older and employed in health care were associated with smaller increases. These associations probably reflect that these prioritized populations were widely vaccinated by the end of March. The Hispanic and Asian population shares were associated with larger increases in vaccination levels. The share of the population without health insurance was associated with smaller increases in vaccination levels.

As we detail in Tables e4.1 and e4.2 in the online supplement, we examined associations stepwise for socioeconomic and racial/ethnic composition. Racial/ethnic composition measures were often statistically significant in the absence of covariates measuring socioeconomic composition. When we included socioeconomic variables; however, the coefficients of the racial/ethnic variables were indistinguishable from zero. We further discuss implications below and in Section e4.3 of the online supplement.

### Simulated Outcomes

**Fig. 2:**
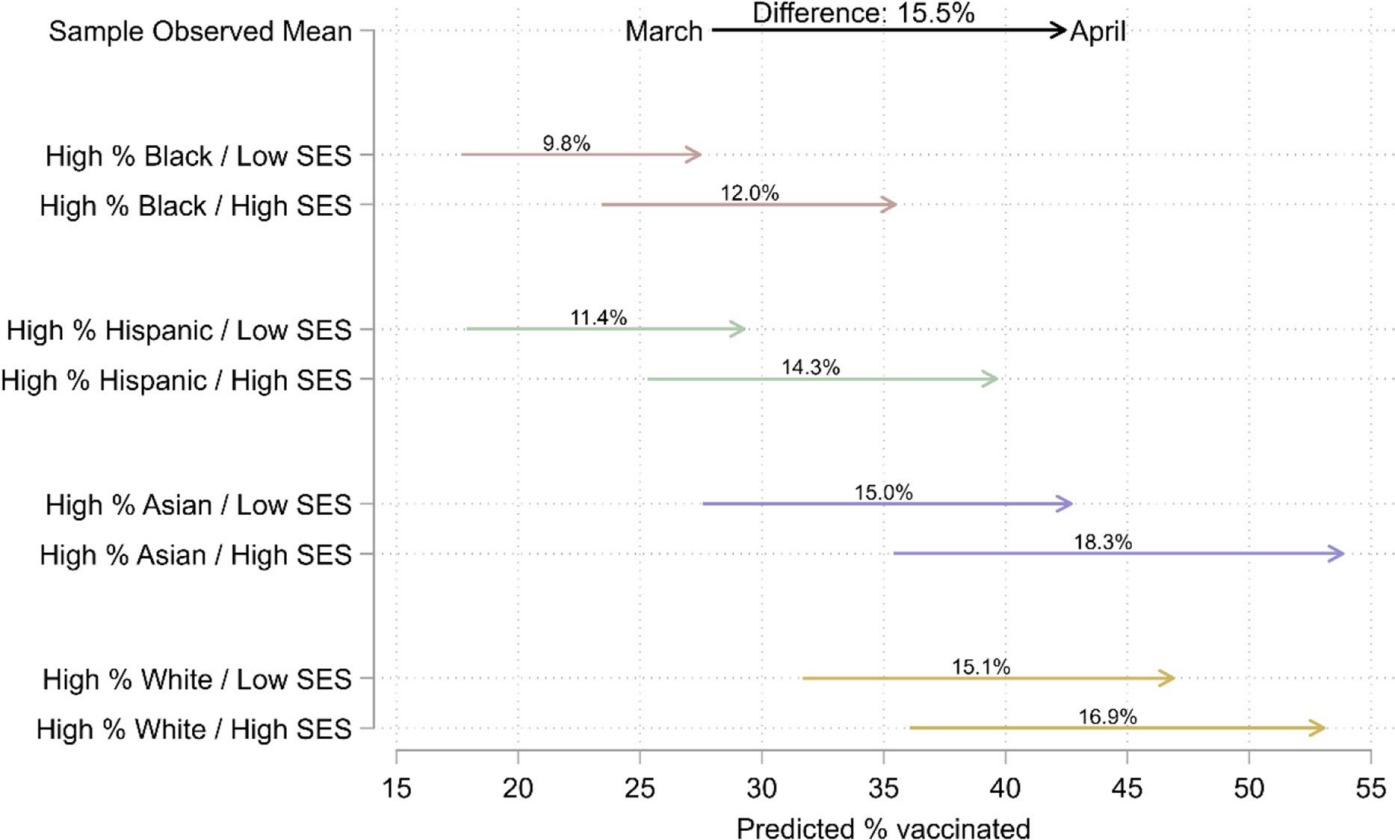
Simulated COVID-19 vaccination levels by racial/ethnic and socioeconomic composition in the population age 15 and older of ZIP Codes across eight large US cities, March and April 2021. Note: This figure illustrates simulated sample-wide means assuming each ZIP Code had a given socioeconomic and racial/ethnic composition. We defined low and high levels as below the 10th and above the 90th within-city percentiles, respectively. We defined SES levels by setting all four socioeconomic variables to the same within-city percentiles within each scenario. We set other independent variables to within-city averages in each scenario. We include the true (observed) sample-wide average values of the dependent variable on the top row for comparison. The “% vaccinated” is the percent of the population age 15 and older with at least one dose of a COVID-19 vaccine

The simulations, illustrated in Fig. 2 and in Fig. e4.2 in the online supplement, contextualize relationships between racial/ethnic and socioeconomic composition. At both time points regardless of racial/ethnic composition, vaccination levels were higher where SES was higher. Socioeconomic disparities in vaccination were smaller where there was a high White population and larger where there were high Black, Hispanic, or Asian populations. In March, the highest vaccination levels (36.1%) were associated with high White populations and high SES; the lowest levels (17.7%) were associated with high Black populations and low SES. In April, the highest vaccination levels (53.8%) were associated with high Asian populations and high SES; the lowest levels (27.5%) were associated with high Black populations and low SES.

Across racial compositions, the simulated change in vaccination levels between March and April was larger where SES was higher, as indicated by the numbers above each line in Fig. 2. Vaccination levels increased most (18.3 p.p.) where there were high Asian populations with high SES, followed by high White populations with high SES (16.9 p.p.). Vaccination levels increased least (9.8 p.p.) where there were high Black populations with low SES, followed by high Hispanic populations with low SES (11.4 p.p.).

## Discussion

### Key Findings

We examined COVID-19 vaccination in eight of the 10 most populous cities in the USA. In March and April 2021, vaccination levels varied more within cities—across ZIP Codes—than between cities. This finding suggests differences in state and local eligibility criteria contributed negligibly to disparities. Our models and simulations confirmed our hypotheses that ZIP Codes with higher shares of POC and low-SES individuals had lower vaccination levels and smaller increases over time. We now turn to three key findings.

Our finding that measures of racial/ethnic composition were statistically insignificant in the presence of socioeconomic covariates does not rule out racial/ethnic disparities. It suggests economic inequality and access to health insurance were fundamental mechanisms of local racial/ethnic gaps in vaccination. Furthermore, the relative magnitudes of the racial/ethnic variables’ coefficients were sometimes nearly as large as those of socioeconomic variables, albeit with slightly larger standard errors. Given the distribution of SES, ZIP Codes with high Black or Hispanic populations were associated with lower vaccination levels than those with high Asian or White populations.

Unlike internet access, measures of health insurance coverage were consistently associated with lower vaccination outcomes. This finding is surprising because internet access but not health insurance was directly tied to obtaining vaccine appointments. The insurance-related variables may capture multiple unobserved mechanisms: unfamiliarity with the medical system, perhaps due to reduced or discriminatory encounters with providers and insurers; incomplete or inaccurate information, including unawareness or skepticism that vaccines were free; and employment or other economic circumstances that impeded getting vaccinated or recovering from side effects. Survey or interview data may clarify individual-level mechanisms. Nonetheless, our results show that residents of large US cities who had tenuous connections to the health care system were less likely to benefit from an intervention that was free to all regardless of insurance coverage.

While several inequalities increased from March to April, one waned. ZIP Codes with high Hispanic populations were associated with larger increases in vaccinations, adjusting for other demographic and socioeconomic factors. Still, accounting for socioeconomic distributions, Hispanic communities were left behind overall as vaccination eligibility expanded.

### Limitations

This study has several limitations. Authorities published vaccination data by ZIP Code only. Because ZIP Codes are suboptimal units for measuring inequality, disparities may be understated in this analysis. Representing ZIP Codes as areal polygons is distortive, potentially leading to measurement error.[145,  146, 147, 148, 149, 150, 151, 152] Furthermore, while they afford more local vantage points than states and counties, ZIP Codes cannot reveal finer, neighborhood-level dynamics. Our units of analysis averaged 38,123 residents, and one-quarter exceeded 50,000. At this scale, observations had substantial within-unit variation and relatively low between-unit variation, likely obscuring disparities.[52, 86, 89, 153, 154, 155, 156, 157, 158] We further discuss the analytical limitations of ZIP Codes in Section e3.1 of the online supplement.

The absence of individual-level data limited this analysis, but geographically aggregated data also presented advantages. It is difficult to determine how much our results reflected differential vaccine eligibility across ZIP Codes. We adjusted for key prioritized populations, however, and by mid-April, eligibility was approaching universal among US adults. In addition, the complete administrative data we used was more comprehensive than small surveys of self-reported behavior. Spatial analysis could also be optimal for guiding policy. Allocating resources geographically may be less resource-intensive than focusing on demographic subgroups. And, as we highlight above, spatial targeting is an effective tool for health equity.

## Conclusion

Even as the number of vaccinated individuals increased by 7.1 million (34.7%) in the large US cities we studied, COVID-19 vaccination lagged in marginalized communities from late March to mid-April 2021. Vaccination gaps increased between low- and high-SES communities and between White or Asian and Black or Hispanic communities. The spatial clustering of unvaccinated individuals probably led to further public health issues.

Our findings suggest vaccination rollouts contributed to cumulative disadvantage at the community—and likely individual—level. Populations that experienced the highest burdens of infection and mortality from COVID-19 before vaccines were available had lower levels of vaccination during restricted vaccine eligibility. Gaps persisted or widened as eligibility first expanded. These disparities may have contributed to a bifurcated recovery in which advantaged communities began to move on from the COVID-19 pandemic while marginalized people continued to suffer.

## Supplementary Information

Below is the link to the electronic supplementary material.

Supplementary file1 (PDF 2.76 MB)
