# Supplementary material for: COVID-19 Vaccine Rollouts and the Reproduction of Urban Spatial Inequality: Disparities Within Large US Cities in March and April 2021 by Racial/Ethnic and Socioeconomic Composition
Source: J Urban Health. 2022 Feb 3;99(2):191–207. doi: 10.1007/s11524-021-00589-0 (PMC8812364; doi:10.1007/s11524-021-00589-0)
Supplement: Supplementary file 1 — Supplementary file1 (PDF 2.76 MB) [file 11524_2021_589_MOESM1_ESM.pdf]

COVID-19 vaccine rollouts and the reproduction of urban spatial inequality: disparities within large US cities in March and April 2021 by racial/ethnic and socioeconomic composition

Online supplement

Nicholas V. DiRago,[a,b] Meiying Li,[c] Thalia Tom,[c]
Will Schupmann,[a] Yvonne Carrillo,[a] Colleen M. Carey,[d]
and S. Michael Gaddis[a,b,*]

[a] Department of Sociology, University of California, Los Angeles (UCLA)
[b] California Center for Population Research, UCLA
[c] Department of Sociology, University of Southern California
[d] Department of Economics, Cornell University
[*]Corresponding author; direct correspondence to mgaddis@soc.ucla.edu.

9 November 2021

e1 Introduction

In this supplement to the main text of our study, "COVID-19 vaccine rollouts and the reproduction of urban spatial inequality: disparities within large US cities in March and April 2021 by racial/ethnic and socioeconomic composition," we clarify our approach and contributions by detailing our data and methods. We used spatial quantitative methods to analyze a novel data set that harmonized previously incompatible administrative, demographic, and geospatial data. These data and methods enabled us to test for socioeconomic and racial/ethnic disparities in COVID-19 vaccination across jurisdictions and reporting agencies.

The main purpose of this supplement is to elaborate on the rationale and decisions that led to the final study. In addition to this document, interested readers can access online code and data to replicate our analysis.[1] We gathered, wrangled, interpolated, and analyzed data using version 4.0.4 of the R statistical programming language and software environment,[2] making extensive use of the Tidyverse.[3] Throughout this supplement, we cite other R packages that we used. We created Figures e3.1 and e4.1 in R[4] and all other figures using version 16.1 of Stata/MP software.[5]

Another purpose of this supplement is to argue that, in addition to the study's empirical findings, our data and methods are substantial contributions. Because of inconsistent reporting, considerable analysis and computation were necessary to compile a high-quality data set measuring local vaccination outcomes across jurisdictions. Once we created the data set, the need to model spatial patterns became evident. Administrative obstacles and empirical dynamics expanded the study's scope beyond the routine boundaries of observational quantitative research.

Even with appropriate adjustments, however, the absence of true neighborhood-level or other hyperlocal data from public sources limited our analysis. Conducted at the ZIP Code level, the study is the best feasible alternative to our original aim of analyzing disparities across jurisdictions by neighborhood—rather than by states or counties, which predominated in media coverage of vaccination rates at the time we began the analysis. Substantial, unnecessary barriers to measuring local inequality in COVID-19 vaccination were in place during the pandemic. Policymakers and administrators should work to reduce them. Reporting data across agencies at the same hyperlocal scale would better support analysis and resource allocation as long as vaccination disparities persist. It would also improve the reporting infrastructure for public health and other sociologically pertinent data.

The rest of this supplement is divided into four sections. In Section e2, we detail the sources, coverage, and definitions of the raw data we collected. We also specify the constructs we sought to measure, define the variables we created accordingly, and assess validity and limitations. In Section e3, we discuss the spatial scale of the study and resulting analytical obstacles and limitations, including measurement error, and detail the interpolation procedures we used to harmonize incompatible raw data. In Section e4, we explain our modeling and estimation strategy, including how we detected and accommodated spatial relationships, and explain the simulation-based approach we used to facilitate interpretation.

Table e2.1: Vaccination data sources and coverage (expanded)

City	Source	As of (year 2021)		Universe
New York	New York City Department of Health and Mental Hygiene	22 March	13 April	New York City residents of MODZCTA
Chicago	Chicago Department of Public Health	22 March	13 April	Chicago residents of ZIP Code
Houston	Texas Department of State Health Services	22 March	11 April	Texas residents of ZIP Code
Phoenix	Arizona Department of Health Services	22 March	13 April	Arizona residents of ZIP Code
Philadelphia	Philadelphia Department of Public Health	21 March	12 April	Philadelphia residents of ZIP Code
San Antonio	Texas Department of State Health Services	22 March	11 April	Texas residents of ZIP Code
San Diego	County of San Diego Health and Human Services Agency	21 March	12 April	San Diego County residents of ZIP Code
Dallas	Texas Department of State Health Services	22 March	11 April	Texas residents of ZIP Code

e2 Data

e2.1 Administrative records

From online public databases maintained by state and local public health authorities, we gathered official counts of individuals with at least one dose of a COVID-19 vaccine in March and April 2021. We secured these data for eight of the 10 most populous U.S. cities: New York, Chicago, Houston, Phoenix, Philadelphia, San Antonio, San Diego, and Dallas (in descending order of population). Suitable data were unavailable for Los Angeles and San José, the second and 10th most populous cities, respectively. We summarize the sources and other details of the vaccination data in Table e2.1.[e]

Only geographically aggregated data were publicly available. For each city except New York, agencies reported vaccination counts aggregated by ZIP Codes (U.S. postal codes) of residence, or by related units known as ZIP Code Tabulation Areas (ZCTAs).[f] For New York, data were aggregated by Modified ZIP Code Tabulation Areas (MODZCTAs), proprietary spatial units that merge less populous ZCTAs with adjacent ZCTAs "to allow more stable estimates of population size for rate calculation."[6] Using an official crosswalk file,[7] we harmonized data released by ZIP Code or ZCTA with the MODZCTAs.

[e] We detail how we interpolated units of analysis that could be meaningfully compared given agencies' diverging reporting practices in Section e3.2.

[f] We further introduce ZCTAs in Section e3.1.

e2.2 Demographic surveys

The 2015–2019 American Community Survey (ACS) Five-Year Estimates were the source of all demographic data in the analysis. Fielded annually by the U.S. Census Bureau (USCB), the ACS provides current, reliable, and representative estimates of population characteristics at various geographic scales.[8] In preparation for the spatial processing detailed in Section e3.2, we collected all ACS tables listed in Table e2.2 by census block group (CBG).[g] We also collected table B01001 by city and by ZCTA.[h] We used the USCB application programming interface (API) to gather the ACS estimates.[9]

We detail the demographic variables we calculated from the ACS Table e2.2.[i] In addition to the denominator for the outcome variables and the population weights, we used ACS data to compute all independent variables, which measured vaccine priority populations, socioeconomic composition, and racial/ethnic composition.

Socioeconomic status (SES) and health care

We conceptualized socioeconomic status (SES) through the conventional sociological lens of life chances,[10,11] or individuals' likelihood of "gaining access to scarce and valued outcomes."[12(p32)] In this analysis, the scarce and valued outcome—COVID-19 vaccination—was health-related and facilitated by internet access. We adjusted for corresponding SES variables, in addition to poverty levels and vaccine priority populations.

Several independent variables measured relationships to the health care system. We partially accounted for the effects of vaccination priority regulations by adjusting for the population of health care workers. Population estimates were unavailable for this exact group, but ACS data allowed us to closely approximate them. We adjusted for the percent of the civilian employed population age 16 or older that worked in "health care and social assistance."[13] This category included employees of hospitals, medical practices, chiropractic practices, dental practices, optometry practices, outpatient and home health care services, nursing and residential care facilities, and other health settings. It also included employees of social service providers, child care services, and other "social assistance" professions. These social assistance workers were typically excluded from early priority groups for vaccination. The population employed in "health care and social assistance" industries was the best available proxy for health care workers, but our variable is effectively an estimate with error.

We also included two independent variables measuring health insurance status. The first was the percent of the population enrolled in Medicaid or other means-tested public health insurance. This variable comprised individuals who had "Medicaid, Medical Assistance, or any kind of government-assistance plan for those with low incomes or a disability."[14p73] It included individuals who had one of these types of insurance in combination with one or more other types of health insurance. The second insurance-related variable was the percent of individuals without health insurance coverage. Together, these two variables captured populations that were among the least integrated into the U.S. health care system.

[g] We introduce CBGs in Section e3.2.

[h] We introduce ZCTAs in Section e3.1.

[i] As the third column of Table e2.2 shows, the ACS tables were sampled from different sub-populations. Variation in the universes was slight and unproblematic for our analysis.

Table e2.2: Variables calculated from 2015–2019 American Community Survey (ACS) Five-Year Estimates

Variable	Table	Universe	Numerator	Denominator
Population	B01001	Total population	Column 1	None
Population age 15 or older	B01001	Total population	Sum of columns 6–25 and 30–49	None
Percent age 65 or older	B01001	Total population	Sum of columns 20–25 and 44–49	Column 1
Percent employed in health care or social assistance	C24030	Civilian employed population age 16 or older	Sum of columns 23 and 50	Column 1
Percent under poverty line	C17002	Population for whom poverty status is determined	Sum of columns 2–3	Column 1
Percent with Medicaid or other means-tested public health insurance	B27010	Civilian non-institutionalized population	Sum of columns 23, 29, 39, 46, and 62	Column 1
Percent without health insurance	B27010	Civilian non-institutionalized population	Sum of columns 33, 50, and 66	Column 1
Percent without internet access	B28002	Households	Column 13	Column 1
Percent Black	B03002	Total population	Column 4	Column 1
Percent Hispanic	B03002	Total population	Column 12	Column 1
Percent Asian	B03002	Total population	Column 6	Column 1
Percent White	B03002	Total population	Column 3	Column 1

We adjusted for income, a key component of SES, by including the percent of the population that was below the poverty line:

> The data on poverty status of households were derived from answers to the income questions. Since poverty is defined at the family level and not the household level, the poverty status of the household is determined by the poverty status of the householder. Households are classified as poor when the total income of the householder's family is below the appropriate poverty threshold. (For nonfamily householders, their own income is compared with the appropriate threshold.) The income of people living in the household who are unrelated to the householder is not considered when determining the poverty status of a household, nor does their presence affect the family size in determining the appropriate threshold. The poverty thresholds vary depending on three criteria: size of family, number of related children, and, for 1- and 2-person families, age of householder.[14p30]

Percent without internet access was an important independent variable because making appointments online was often the most effective way to secure a vaccine. This variable was the only variable measured at the household level. In the ACS, internet access is defined as "whether or not someone in the household uses or can connect to the internet, regardless of whether or not they pay for the service."[14p13] Households are designated as having internet access if at least one member can access the internet through a computer or mobile device.

Race/ethnicity

We use the term "race/ethnicity" rather than "race," "ethnicity," or "race and ethnicity." In the 2015–2019 ACS, USCB considered Hispanic, Latino, and Spanish origins as indicators of ethnicity and other origins as indicators of race.[14] USCB racial/ethnic definitions change over time and are often unaligned with popular understandings of race/ethnicity, academic definitions of race/ethnicity, or analytically appropriate racial/ethnic schemes for a research question or site.[15–22] A sharp distinction between race and ethnicity does not reflect the processes of stratification in which we were interested in this analysis. The combined term "race/ethnicity" communicates the construct of interest and the structure of the data we used to measure it.

From ACS data,[8,14] we created variables measuring the estimated populations of four mutually exclusive, non-exhaustive racial/ethnic groups: Black, Hispanic, Asian, and White. We defined Hispanic as Hispanic, Latino, or Spanish origin, of any race(s). We defined Black, Asian, and White as Black or African American alone, Asian alone, and White alone, respectively, and non-Hispanic. This approach implies a fifth category comprised of non-Hispanic individuals of multiple races or of any other race alone, including American Indians, native Alaskans and Hawaiians, and Pacific Islanders. The four racial/ethnic variables did not sum to one (100 percent) unless the estimated population of the fifth category was zero.

Thoroughly accounting for limitations stemming from USCB racial/ethnic categories was outside the scope of our analysis, but attending to the definitions of racial/ethnic categories aids in interpreting our findings. In the ACS,

> The terms "Hispanic," "Latino," and "Spanish" are used interchangeably. Some respondents identify with all three terms while others may identify with only

> one of these three specific terms. Hispanics or Latinos who identify with the terms "Hispanic," "Latino," or "Spanish" are those who classify themselves in one or more of the specific Hispanic, Latino, or Spanish categories listed on the questionnaire ("Mexican," "Puerto Rican," or "Cuban") as well as those who indicate that they are "another Hispanic, Latino, or Spanish origin." ... People who identify their origin as Hispanic, Latino, or Spanish may be of any race.[14p76]

The ACS classifies individuals as White if they report "origins in any of the original peoples of Europe, the Middle East, or North Africa," including people who "report entries such as Irish, German, Italian, Lebanese, Arab, Moroccan, or Caucasian."[14p114] It classifies individuals as Black or African American if they report "origins in any of the Black racial groups of Africa," including people who "report entries such as African American, Kenyan, Nigerian, or Haitian."[14p114] It classifies individuals as Asian if they report "origins in any of the original peoples of the Far East, Southeast Asia, or the Indian subcontinent including, for example, Cambodia, China, India, Japan, Korea, Malaysia, Pakistan, the Philippine Islands, Thailand, and Vietnam."[14p115]

e2.3 Geospatial data

We used several geospatial vector datasets from the USCB 2019 TIGER/Line Shapefiles (TLS). The TLS are extracts of official USCB geographic and cartographic data.[23] We collected the following TLS data from the USCB API:[24] city boundaries from the Place State-Based Shapefiles; ZCTA boundaries from the Five-Digit ZCTA National Shapefile; CBG boundaries from the Block Group State-Based Shapefiles; the boundaries of bodies of water from the Area Hydrography County-Based Shapefiles; and the boundaries of USCB-designated landmarks from the Area Landmark State-Based Shapefiles. The coordinate reference system (CRS) for the TLS data was the North American Datum of 1983 (NAD83), an ellipsoidal system that uses geodetic latitude and longitude (not a Cartesian plane).

e3 Spatial processing

e3.1 ZIP Codes, ZIP Code Tabulation Areas (ZCTAs), and vaccination records

For brevity and interpretability, in the main paper we refer to our units of analysis as ZIP Codes, the official and colloquial name for postal codes in the U.S. ZIP Codes were the bases of the units of analysis but themselves were not viable analytical units. ZIP Codes are lists of discrete postal addresses, not areal units. More specifically, they are

> administrative units established by the United States Postal Service (USPS) for the distribution of mail. ZIP Codes serve addresses for the most efficient delivery of mail, and therefore generally do not respect political or census statistical area boundaries. ZIP Codes usually do not have clearly identifiable boundaries, often serve a continually changing area, are changed periodically to meet postal requirements, and do not cover all the land area of the United States.[25pA–13]

Table e3.1: Population distribution of ZIP Code Tabulation Areas (ZCTAs) within and across eight large U.S. cities, 2015–2019

City	ZCTAs	Population				Area				Population density			
		M	*SD*	10%	90%	*M*	*SD*	10%	90%	*M*	*SD*	10%	90%
New York	196	44,673	27,176	9,151	83,574	4.4	4.2	0.9	8.2	16,237	12,669	3,209	35,127
Chicago	87	40,687	25,148	11,559	79,690	11.8	8.5	2.6	20.2	4,825	3,617	1,378	9,804
Houston	145	37,194	21,173	16,170	59,835	43.9	60.7	9.7	97.3	1,518	968	410	2,789
Phoenix	67	36,025	17,375	10,064	60,657	52.1	85.6	10.4	107.5	1,500	1,004	255	2,794
Philadelphia	68	28,603	18,284	6,158	53,988	9.6	7.8	3.3	17.6	4,269	3,163	863	8,371
San Antonio	69	28,439	19,865	4,818	56,665	49.1	50.1	5.8	106.7	1,043	777	74	2,092
San Diego	58	38,047	22,312	4,431	64,465	34.6	59.8	7.9	59.7	1,921	1,267	432	3,764
Dallas	86	33,483	19,757	8,188	56,424	33.1	34.7	8.8	67.4	1,523	955	467	2,629
**Overall**	**776**	**37,495**	**23,203**	**8,754**	**69,876**	**26.6**	**47.8**	**2.1**	**57.5**	**5,835**	**9,016**	**495**	**16,875**

Includes the total population and geographic area of all ZCTAs that intersected the cities proper.
Area measured in square kilometers; corresponds to A_i in Equation e3.2.

We operationalized ZIP Codes as ZCTAs. For each ZIP Code, the USCB delimits a corresponding ZCTA that approximates the ZIP Code as a polygonal areal unit. The area of each ZCTA is mutually exclusive with those of all other ZCTAs.[23] ZIP Codes and ZCTAs are related and frequently used interchangeably in research, but ZCTAs are suboptimal units for spatial and quantitative analysis.

ZCTAs are distortive and error-prone. ZIP Codes do not have objective, non-overlapping areal boundaries, which often makes converting from ZIP Codes to ZCTAs uncertain. Public health scholars, epidemiologists, and spatial analysts consistently find mismatches between observations' true locations and recorded ZCTAs.[26–29] Despite the well-known risk of inducing measurement error, however, most state and local authorities published COVID-19 vaccination data aggregated by ZIP Code. Some failed to indicate whether data were aggregated by ZIP Code or ZCTA. These reporting decisions left researchers without a feasible alternative to using ZCTAs to define areal boundaries consistently across cities.

We summarize the population distribution of each ZCTA that intersected the eight cities proper in Table e3.1. The average ZCTA had 37,495 estimated inhabitants and spanned 26.6 square kilometers, an area nearly half the size of Manhattan or roughly twice the size of Los Angeles International or London Heathrow Airports. In addition, ZCTAs' physical and population sizes varied widely. Over 20 percent of ZCTAs had fewer than 10,000 or more than 70,000 estimated inhabitants. Some were as small as two square kilometers, just over half the size of Central Park in Manhattan; others approached or exceeded 60 square kilometers, an area larger than that of Manhattan and about one-third the size of Washington, DC.

ZCTAs have much larger geographical areas and populations than colloquial and academic definitions of neighborhoods, which limits their utility for studying inequality. Using ZCTAs as units of analysis complicates making connections with the voluminous literature on neighborhood effects,[30,31] which usually uses finer spatial units. For example, census tracts—the most common areal units in analyses of neighborhood inequality in the U.S.—average 4,000 residents and rarely have more than 8,000.[8,32] ZIP Codes and ZCTAs also differ substantially from subjective neighborhood boundaries,[33–37] activity spaces,[38–41] and other, analytically sounder local units.[42–47] Aggregating person-level observations to a scale as wide as ZCTAs increases variation within units and decreases variation between them. Statistical analysis may therefore mischaracterize disparities or fail to detect them al-

together.[48–53] For these reasons, we avoid the term "neighborhood" when describing our units and findings, and our study's evidence of inequality is partial and potentially understated.

Issues of spatial mismatch and scale aside, ZIP Codes and ZCTAs are inconvenient reporting units for comparing outcomes across administrative jurisdictions. ZIP Codes and ZCTAs overlap neighborhoods, municipalities, counties, and states.[8] As the rightmost column of Table e2.1 shows, the jurisdictions of some agencies that reported vaccination data also overlapped political boundaries, frequently excluding part of the territory of some ZCTAs. Several sources of data extended only to the populations of cities proper, excluding vaccinations administered to residents of other municipalities who lived in ZIP Codes that straddled city borders. Other agencies' vaccination counts included all individuals with a given ZIP Code, regardless of the municipality in which they resided, within a particular state or county. The administrative and demographic data required adjustment before we could meaningfully compare trends among units.

e3.2 Spatial interpolation

To create consistent, comparable units of analysis given reporting irregularities in the vaccination data, we restricted the analysis to the geographical limits of the eight cities proper. We excluded populations residing outside the cities, even if they shared a ZIP Code with some city residents. Because only aggregated administrative and demographic data were available, we relied on spatial interpolation to approximate the within-city values of variables for ZCTAs that spanned city borders.

We used the overlay or areal weighting method of spatial interpolation.[54,55] This method estimates the value of a variable in a target zone based on the proportion of smaller source zones that intersect it. Formally,

$$\tilde{C}_t = \sum_s C_s \frac{A^*_{s\cap t}}{A^*_s}, \tag{e3.1}$$

where C was the observed value of a count variable; $\tilde{C}$ was the interpolated value of a count variable; A^*_i approximated the geographic area of unit i that could be populated; $\cap$ denoted geometric intersection; and s and t denoted the source and target zones, respectively. We conducted the interpolation using the NAD83 CRS and square kilometers as the areal unit of measure.[56,57]

The target zones were the geometric intersections between 776 ZCTAs and the eight cities proper. (For ZCTAs that were completely inside city limits, the target zone was the entire ZCTA). The source zones were 16,283 CBGs that intersected the cities proper. The most local areal units for which the USCB releases ACS estimates, CBGs' populations typically range from 600 to 3,000 people.[8] The area of 90% of CBGs that intersected the cities was 1.33 square kilometers or less. The typical CBG was 0.93 square kilometers—less than four percent of the area of the typical ZCTA in the study.

The overlay method had several benefits for this study. It also entailed one nontrivial assumption. Overlay interpolation accounts for uneven population density within target zones at the level of source zones. In addition, it is intuitive, computationally inexpensive, and does not require supplemental data. Using the overlay method required us to assume, however,

Table e3.2: Population distribution of census block groups (CBGs) within and across eight large U.S. cities, 2015–2019

City	CBGs	Population				Area				Population density			
		M	*SD*	10%	90%	*M*	*SD*	10%	90%	*M*	*SD*	10%	90%
New York	6,507	1,305	692	615	2,136	0.14	0.53	0.02	0.23	24,284	21,247	4,479	52,754
Chicago	2,344	1,246	642	599	1,992	0.32	0.81	0.09	0.50	7,970	9,083	2,064	13,989
Houston	1,951	2,342	2,796	844	3,701	2.32	6.84	0.31	4.03	2,629	2,601	639	5,012
Phoenix	1,064	1,705	815	876	2,714	2.17	11.45	0.32	2.61	2,612	1,980	626	5,045
Philadelphia	1,402	1,185	585	555	1,963	0.33	0.91	0.06	0.55	8,645	5,755	2,223	16,333
San Antonio	1,007	1,796	1,213	759	3,153	2.68	9.95	0.35	3.66	2,098	1,304	574	3,731
San Diego	924	1,789	1,572	764	2,750	1.95	10.37	0.16	1.90	4,125	3,307	946	8,029
Dallas	1,084	1,553	927	711	2,584	1.48	3.40	0.21	2.68	2,987	3,078	627	6,173
**Overall**	**16,283**	**1,511**	**1,296**	**657**	**2,462**	**0.93**	**5.32**	**0.04**	**1.33**	**12,644**	**17,088**	**1,064**	**33,315**

Includes the total population and geographic area of all CBGs that intersected the cities proper.
Area measured in square kilometers; corresponds to A_i in Equation e3.2.

that populations were uniformly distributed within CBGs. This assumption was modest for such small units.

We nonetheless took steps to mitigate potential inaccuracies. As Equation e3.1 indicates, we interpolated $\tilde{C}_t$ from A^* rather than from A. Formally,

$$A_i^* = A_i - A_{i \cap (B \cup L)} \tag{e3.2}$$

where A_i was the total area of unit i; $\cup$ denoted geometric union; B was the set of USCB-designated bodies of water; and L was a subset of USCB-designated landmarks. B and L overlapped in many locations. B included the area of "perennial and intermittent ... ponds, lakes, oceans, swamps, glaciers, and ... large streams."[23p3-34] We list the types of landmarks included in L in Table e3.3. By excluding from areal calculations parts of source zones that were unlikely to contribute population to the target zones, the adjustment in Equation e3.2 made it more plausible to assume uniform population density within CBGs.

We visualize the overlay interpolation process in Figure e3.1. We used overlay interpolation to estimate the target zones' numerators and denominators for all variables listed in Table e2.2—including the population age 15 and older, the outcome variable's denominator.

Calculating the numerator for the outcome variable—the number of individuals with at least dose of a COVID-19 vaccine—required a separate interpolation procedure. For New York, Chicago, and Philadelphia, agencies' counts included only vaccinated individuals living inside the cities proper. In these cases, we could adopt the reported total directly as the numerator for the outcome variable. Elsewhere, reporting agencies' counts for ZIP Codes intersecting the cities included vaccinated individuals residing outside the cities proper. We resolved this discrepancy by adjusting the reported counts using multipler m. The value of m was one for observations in New York, Chicago, and Philadelphia; otherwise, it was the ratio between the estimated populations of the target zone and its corresponding reporting unit. Formally,

Table e3.3: U.S. Census Bureau (USCB)-designated landmarks excluded from area calculations

Code	Description
C3026	Quarry or mine
C3077	Solar farm
K2180	Park
K2181	National park, forest, etc.
K2182	Other federal land
K2183	Tribal park, forest, etc.
K2184	State park, forest, etc.
K2185	Regional park, forest, etc.
K2186	County park, forest, etc.
K2187	Civil division park, forest, etc.
K2188	Municipal park, forest, etc.
K2189	Private park, forest, etc.
K2190	Other park, forest, etc.
K2362	Industrial building or park
K2424	Marina
K2432	Pier or dock
K2451	Airport
K2452	Train station
K2453	Bus terminal
K2454	Marine terminal
K2455	Seaplane anchorage
K2456	Airport terminals
K2457	Airport grounds
K2458	Park-and-ride facility
K2459	Airport runway
K2460	Helicopter landing
K2561	Golf course
K2564	Amusement park
K2582	Cemetery
K2586	Zoo

Codes are USCB MAF/TIGER Feature Class Codes[23].

Figure e3.1: Summary of overlay interpolation by city

New York

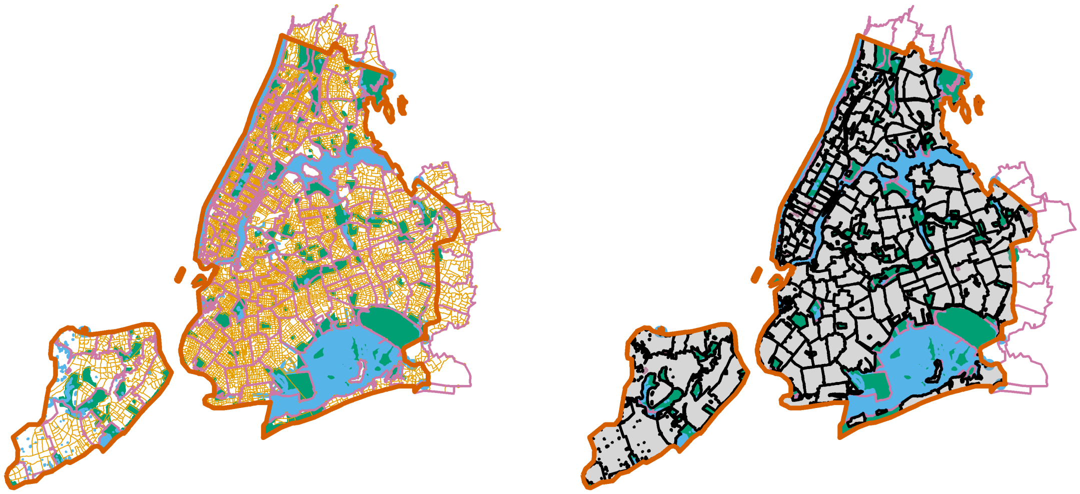

Chicago

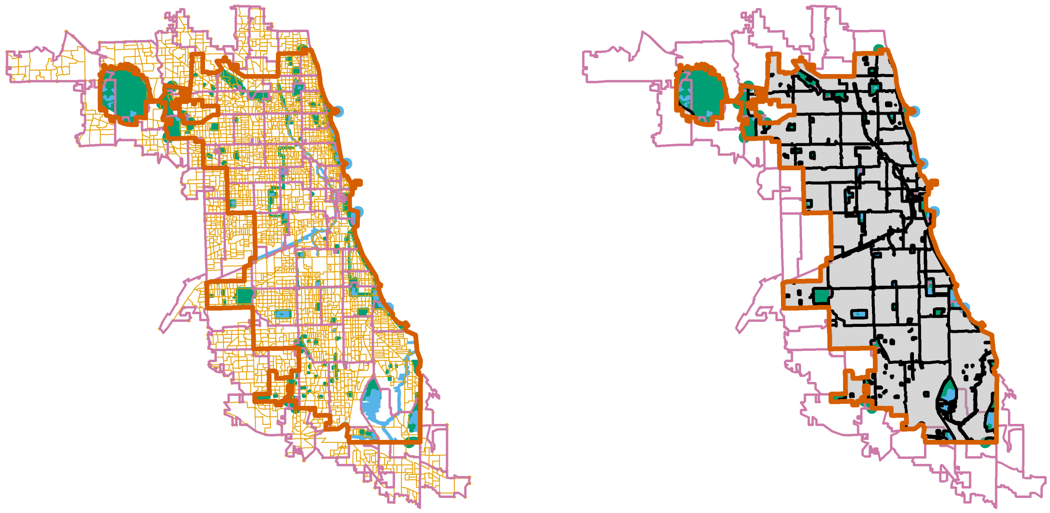

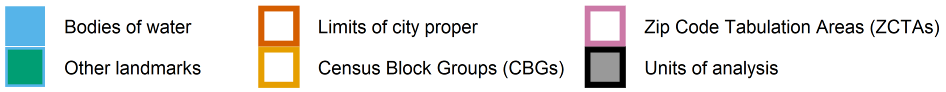

Scales differ. All ZCTAs that intersect cities shown.

Figure e3.1: Summary of overlay interpolation by city

Houston

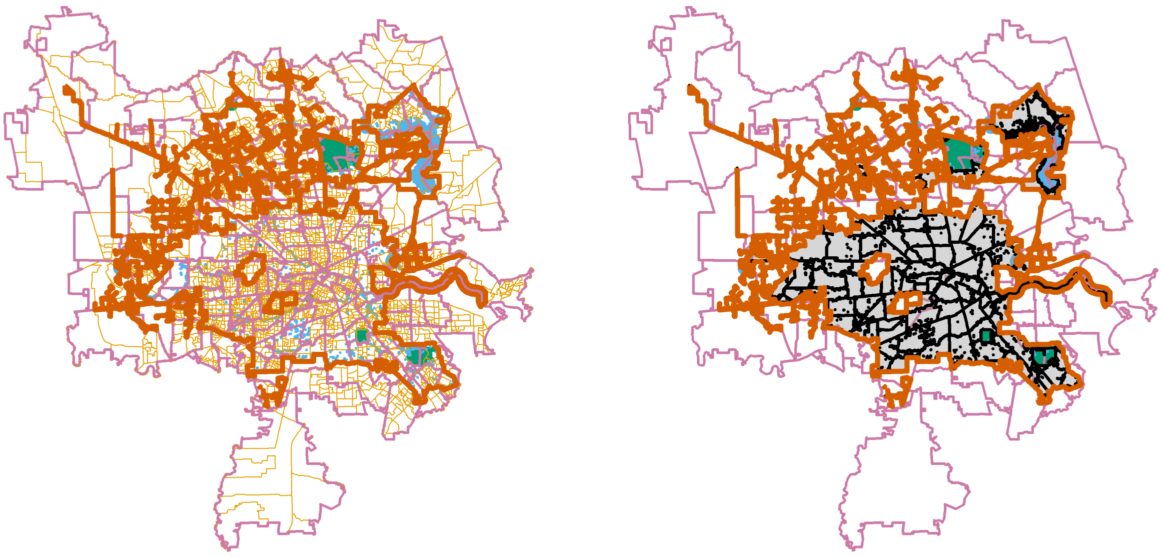

Phoenix

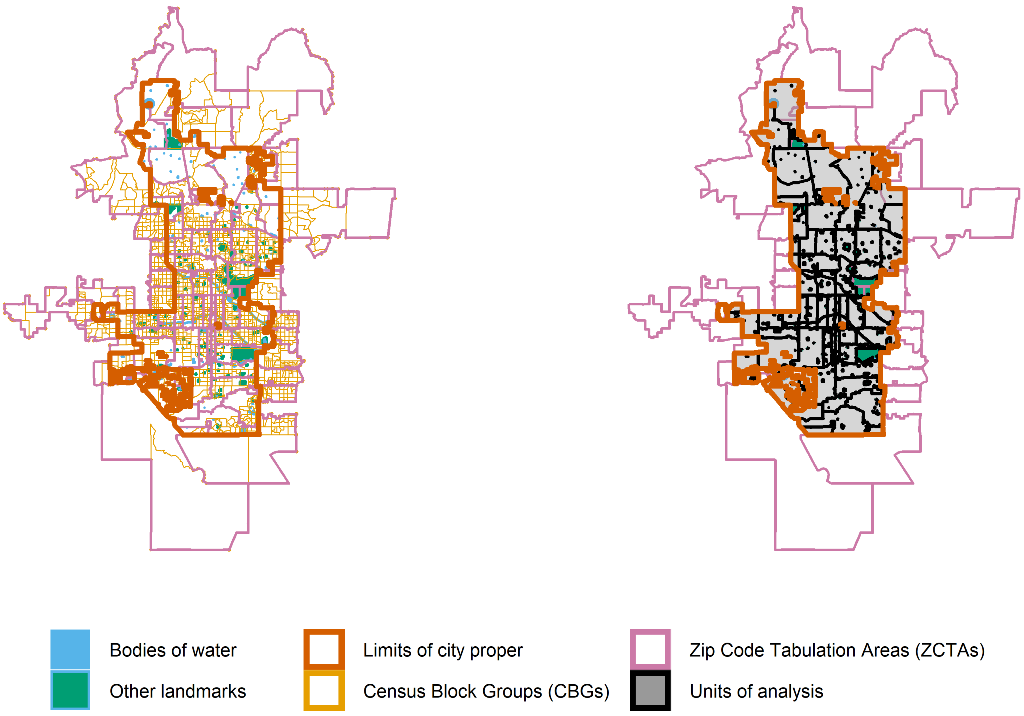

Scales differ. All ZCTAs that intersect cities shown.

Figure e3.1: Summary of overlay interpolation by city

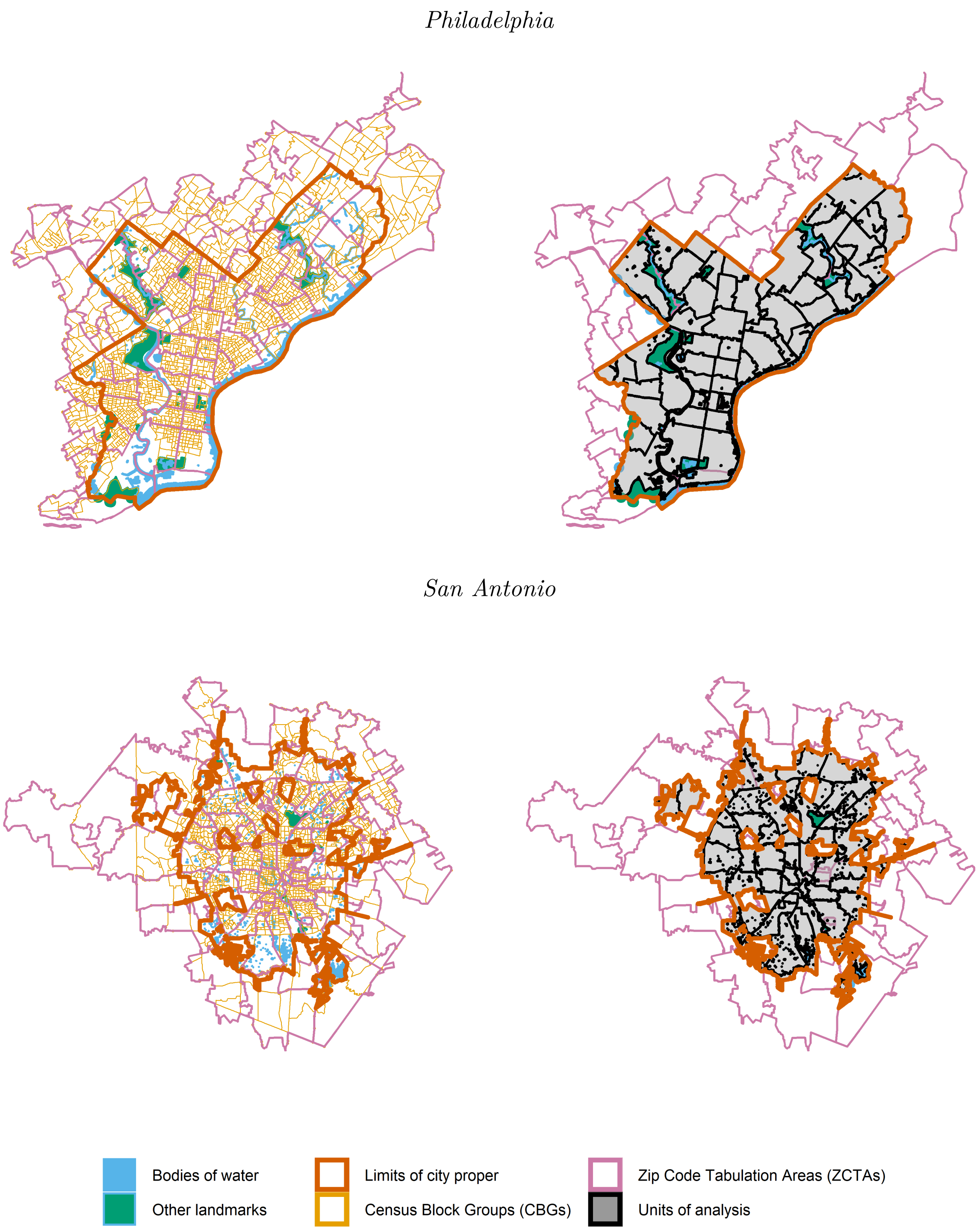

Scales differ. All ZCTAs that intersect cities shown.

Figure e3.1: Summary of overlay interpolation by city

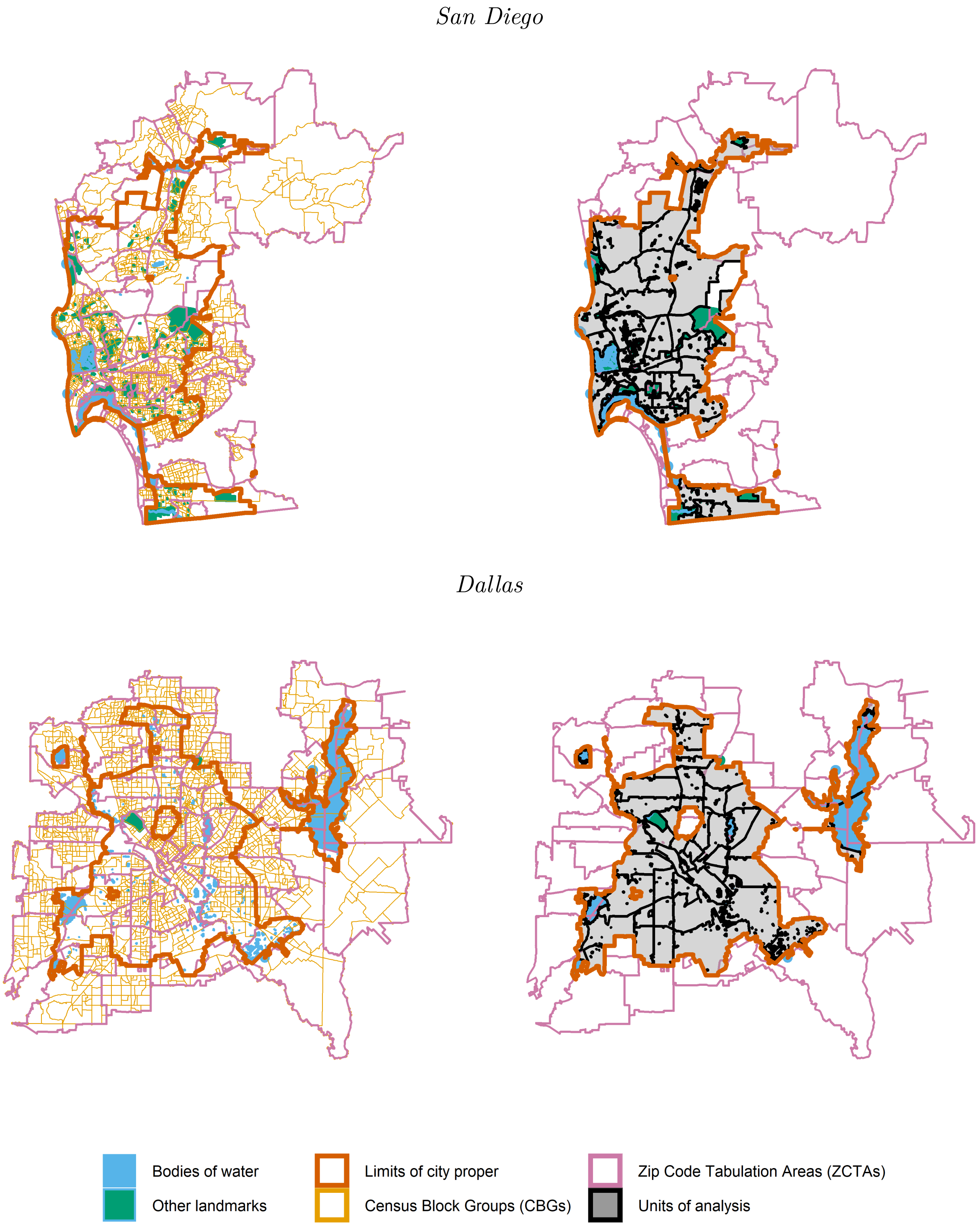

Scales differ. All ZCTAs that intersect cities shown.

Table e3.4: Population distribution of interpolated units of analysis within and across eight large U.S. cities, 2015–2019

City	Units	Population				Area				Population density			
		M	*SD*	10%	90%	*M*	*SD*	10%	90%	*M*	*SD*	10%	90%
New York	175	48,078	26,244	18,050	84,458	3.6	3.3	1.0	6.2	18,864	12,726	5,443	36,182
Chicago	53	51,131	24,084	21,882	86,091	9.7	8.3	2.2	16.6	7,376	4,215	3,271	13,798
Houston	99	24,998	15,270	6,933	43,964	16.0	9.1	5.4	28.4	1,708	928	745	2,830
Phoenix	50	32,331	17,292	7,825	53,528	25.7	20.6	5.6	54.0	1,707	990	360	3,035
Philadelphia	46	34,328	17,463	12,436	59,943	7.1	4.3	2.4	13.6	5,965	2,899	2,295	9,673
San Antonio	48	31,560	17,439	6,984	56,254	25.9	15.4	7.1	48.1	1,392	704	482	2,360
San Diego	33	42,400	18,163	22,666	67,742	22.1	16.4	8.8	46.8	2,603	1,487	947	4,690
Dallas	48	27,830	20,596	4,760	56,793	18.2	15.5	4.1	38.7	1,857	1,035	734	3,592
**Overall**	**552**	**38,123**	**23,314**	**9,166**	**72,696**	**13.0**	**13.8**	**1.7**	**31.1**	**8,085**	**10,555**	**901**	**22,545**

Area measured in square kilometers; corresponds to A_i^* in Equation e3.2.

$$\tilde{v}_t = m_t v_z, \tag{e3.3}$$

$$m_t = \begin{cases} 1 & z_t \equiv t \\ \tilde{q}_t / q_{z_t} & z_t \not\equiv t, \end{cases} \tag{e3.4}$$

where v_i and $\tilde{v}_i$ were the observed and interpolated numbers of individuals with at least one vaccine dose in unit i, respectively; q_i and $\tilde{q}_i$ were the observed and interpolated total populations of unit i, respectively; and z_t denoted the area covered by the reporting unit corresponding to target zone t. This adjustment assumed that, within ZCTAs that straddled city borders, vaccinated individuals were distributed proportionally to the total population.

We made two final adjustments to the interpolated units of analysis. The interpolation yielded several target zones with very small estimated populations. To minimize the effects of outliers and reduce variation in the size of the units, we merged target zones with unusually low populations into adjacent zones such that no unit of analysis had fewer than 3,000 estimated residents. In addition, in a small number cases, implausible or extreme values surfaced in the interpolated vaccination outcome variables. These values suggested spatial mismatch occurred in the ZIP Code-level data, or that the interpolation was particularly ill-suited to the spatial contours of a portion of the study area. To smooth out mismatches and discrepancies in these instances, we merged sets of contiguous zones that visual inspection suggested were susceptible to administrative mismatch or inaccurate interpolation. Due to these adjustments, there were 776 target zones but only 552 final units of analysis.

We summarize the population distribution of the 552 ZIP Code-based units of analysis in Table e3.4, and we illustrate them in the right-hand panels of Figure e3.1. The average unit of analysis had an interpolated total population of 38,123, roughly equivalent to the average population of the ZCTAs that were the bases of the target zones. Units' populations varied considerably. About one-quarter had more than 50,000 estimated inhabitants; another quarter had fewer than 20,000. Because the units excluded bodies of water, landmarks, and area outside city limits, however, the average physical size of the units of analysis was roughly half that of the average ZCTA. Furthermore, the area of the units of analysis exhibited significantly less variation than the raw ZCTAs. On the whole, the interpolated spatial units

Table e3.5: Official vs. interpolated estimates of the populations of eight large U.S. cities, 2015–2019

	Official	Interpolated	Difference
New York	8,419,316	8,413,650	−0.1%
Chicago	2,709,534	2,709,943	+0.0%
Houston	2,310,432	2,474,802	+7.1%
Phoenix	1,633,017	1,616,550	−1.0%
Philadelphia	1,579,075	1,579,088	+0.0%
San Antonio	1,508,083	1,514,880	+0.5%
San Diego	1,409,573	1,399,200	−0.7%
Dallas	1,330,612	1,335,840	+0.4%
**Overall**	**20,899,642**	**21,043,953**	**+0.7%**

Official estimates from ACS (2015–2019) table B01001, column 1.

provided more viable bases for analysis and comparison than were available in the raw data. Yet they generally remained susceptible to the analytical pitfalls of ZIP Codes and ZCTAs that we discuss in Section e3.1. We present descriptive statistics for observations on the analysis variables for the interpolated units of analysis in Table e3.6.

e3.3 Measurement error and information bias

In regression analysis, information bias arises when variables are measured inaccurately.[58] If outcome variables are measured with error, regression coefficients are unbiased but confidence intervals too wide.[59,60] Researchers might consequently accept null hypotheses they should reject. If independent variables are measured with error, the direction of information bias is case-specific, determined by the covariance of the variables in question.[59,61] Typically, regression coefficients are biased towards zero when independent variables are measured with error.[60,62] As a result, researchers are vulnerable to understating the magnitude of associations. Measurement error often makes regression inference more conservative.

The nature of available data for this study made some information bias likely. The demographic data were estimates with margins of error.[8] The administrative records were vulnerable to spatial mismatch between individuals' ZIP Codes and assigned ZCTAs. Even careful variable construction from these data also probably introduced some measurement error because available quantities were not always aligned with the theoretical quantity of interest. Similarly, as an estimation process based on aggregated observations, overlay interpolation contributed some measurement error.

It is unlikely that these sources of information bias seriously jeopardized our findings. The direction and magnitude of bias from measurement error are difficult to predict, but information bias probably only made our analysis more cautious. The quality of the data was high overall. We used established methods to calculate variables that tracked closely with theoretical variables of interest. Moreover, because information bias typically makes regression findings more conservative, false negatives (Type II errors) were more likely than false positives (Type I errors). The most plausible effect of information bias in this study

Table e3.6: Descriptive statistics on COVID-19 vaccination and population composition in interpolated units of analysis within and across eight large U.S. cities, March and April 2021 (expanded)

City	Variable	*M*	*SD*	10%	90%
New York ($n = 175$)	Percent age 15 or older with at least one dose, March	28.2%	7.9%	18.8%	37.2%
	Percent age 15 or older with at least one dose, April	43.6%	11.7%	29.4%	59.1%
	Percent age 15 or older with at least one dose, difference	15.4%	5.2%	9.7%	22.9%
	Percent age 65 or older	15.0%	5.1%	9.4%	21.5%
	Percent employed in health care or social assistance	17.4%	6.5%	9.5%	26.5%
	Percent under poverty line	15.9%	9.4%	6.2%	29.7%
	Percent with Medicaid or other means-tested public health insurance	21.8%	12.3%	6.8%	40.9%
	Percent without health insurance	8.2%	4.4%	3.1%	13.6%
	Percent without internet access	14.6%	6.5%	6.5%	23.2%
	Percent Black	19.8%	23.4%	1.2%	58.2%
	Percent Hispanic	26.4%	19.3%	8.1%	61.3%
	Percent Asian	14.8%	14.0%	1.9%	35.3%
Chicago ($n = 53$)	Percent age 15 or older with at least one dose, March	28.9%	5.5%	21.3%	34.2%
	Percent age 15 or older with at least one dose, April	45.9%	9.7%	31.0%	58.0%
	Percent age 15 or older with at least one dose, difference	17.0%	5.5%	9.9%	24.2%
	Percent age 65 or older	12.5%	4.1%	6.8%	18.1%
	Percent employed in health care or social assistance	13.9%	3.7%	10.3%	19.7%
	Percent under poverty line	17.9%	9.9%	7.8%	32.4%
	Percent with Medicaid or other means-tested public health insurance	16.2%	11.1%	2.9%	30.8%
	Percent without health insurance	10.1%	6.0%	3.0%	17.8%
	Percent without internet access	16.3%	9.1%	3.5%	29.5%
	Percent Black	29.7%	33.5%	1.7%	89.3%
	Percent Hispanic	22.4%	21.9%	3.2%	52.7%
	Percent Asian	7.9%	8.6%	0.2%	19.4%
Houston ($n = 99$)	Percent age 15 or older with at least one dose, March	27.2%	10.0%	17.9%	39.9%
	Percent age 15 or older with at least one dose, April	41.0%	13.6%	28.1%	59.5%
	Percent age 15 or older with at least one dose, difference	13.7%	4.0%	9.6%	19.9%
	Percent age 65 or older	10.3%	3.1%	6.6%	14.6%
	Percent employed in health care or social assistance	10.8%	3.3%	7.3%	15.5%
	Percent under poverty line	18.3%	9.4%	6.9%	32.8%
	Percent with Medicaid or other means-tested public health insurance	9.0%	5.5%	2.5%	17.7%
	Percent without health insurance	25.1%	12.3%	8.8%	40.8%
	Percent without internet access	16.5%	10.9%	4.5%	31.2%
	Percent Black	22.7%	18.6%	3.7%	48.8%
	Percent Hispanic	42.6%	22.3%	18.0%	75.2%
	Percent Asian	6.6%	6.2%	0.6%	15.1%

Table e3.6: Descriptive statistics on COVID-19 vaccination and population composition in interpolated units of analysis within and across eight large U.S. cities, March and April 2021 (expanded)

City	Variable	*M*	*SD*	10%	90%
Phoenix ($n = 50$)	Percent age 15 or older with at least one dose, March	27.8%	11.0%	14.3%	43.9%
	Percent age 15 or older with at least one dose, April	40.2%	13.5%	24.5%	58.2%
	Percent age 15 or older with at least one dose, difference	12.4%	3.2%	9.1%	16.1%
	Percent age 65 or older	11.3%	4.3%	6.2%	16.4%
	Percent employed in health care or social assistance	12.1%	2.2%	9.0%	14.7%
	Percent under poverty line	16.8%	10.0%	4.7%	31.5%
	Percent with Medicaid or other means-tested public health insurance	15.2%	8.3%	4.6%	27.2%
	Percent without health insurance	14.4%	8.3%	4.6%	26.7%
	Percent without internet access	13.5%	9.4%	2.6%	26.6%
	Percent Black	6.0%	4.3%	2.0%	12.3%
	Percent Hispanic	37.3%	24.0%	10.2%	72.5%
	Percent Asian	4.0%	2.8%	0.9%	7.7%
Philadelphia ($n = 46$)	Percent age 15 or older with at least one dose, March	23.5%	7.6%	15.5%	35.1%
	Percent age 15 or older with at least one dose, April	35.6%	8.7%	25.7%	48.3%
	Percent age 15 or older with at least one dose, difference	12.1%	2.1%	9.6%	14.6%
	Percent age 65 or older	14.2%	4.6%	8.5%	20.5%
	Percent employed in health care or social assistance	20.6%	4.4%	14.8%	27.4%
	Percent under poverty line	22.3%	11.2%	10.0%	38.7%
	Percent with Medicaid or other means-tested public health insurance	19.9%	11.3%	5.9%	34.8%
	Percent without health insurance	8.7%	3.6%	4.7%	13.5%
	Percent without internet access	18.2%	8.5%	7.9%	27.1%
	Percent Black	38.5%	31.0%	5.7%	84.1%
	Percent Hispanic	12.0%	13.5%	2.9%	28.7%
	Percent Asian	7.0%	5.8%	1.1%	14.7%
San Antonio ($n = 48$)	Percent age 15 or older with at least one dose, March	29.1%	9.0%	21.1%	44.5%
	Percent age 15 or older with at least one dose, April	42.2%	11.9%	32.3%	60.7%
	Percent age 15 or older with at least one dose, difference	13.1%	3.3%	10.2%	17.6%
	Percent age 65 or older	11.9%	3.2%	8.4%	15.3%
	Percent employed in health care or social assistance	14.1%	2.3%	12.1%	17.2%
	Percent under poverty line	16.5%	8.9%	4.4%	27.1%
	Percent with Medicaid or other means-tested public health insurance	8.3%	5.1%	2.6%	15.3%
	Percent without health insurance	18.9%	8.4%	6.9%	29.6%
	Percent without internet access	15.7%	9.9%	3.6%	29.0%
	Percent Black	7.1%	7.7%	0.9%	17.8%
	Percent Hispanic	61.8%	20.9%	34.6%	89.8%
	Percent Asian	2.9%	2.7%	0.4%	6.8%

Table e3.6: Descriptive statistics on COVID-19 vaccination and population composition in interpolated units of analysis within and across eight large U.S. cities, March and April 2021 (expanded)

City	Variable	M	SD	10%	90%
San Diego ($n = 33$)	Percent age 15 or older with at least one dose, March	34.2%	8.4%	26.6%	43.8%
	Percent age 15 or older with at least one dose, April	50.3%	10.8%	40.0%	66.8%
	Percent age 15 or older with at least one dose, difference	16.1%	3.5%	13.4%	21.3%
	Percent age 65 or older	13.2%	4.4%	9.0%	19.7%
	Percent employed in health care or social assistance	12.9%	2.0%	10.2%	15.2%
	Percent under poverty line	12.0%	6.7%	5.2%	23.0%
	Percent with Medicaid or other means-tested public health insurance	12.3%	8.8%	4.4%	23.9%
	Percent without health insurance	8.1%	5.4%	2.8%	15.3%
	Percent without internet access	7.0%	5.3%	1.6%	13.3%
	Percent Black	5.3%	4.4%	1.4%	11.3%
	Percent Hispanic	26.8%	21.4%	9.1%	58.0%
	Percent Asian	15.4%	11.4%	4.3%	30.3%
Dallas ($n = 48$)	Percent age 15 or older with at least one dose, March	27.0%	10.4%	16.1%	42.9%
	Percent age 15 or older with at least one dose, April	42.0%	13.9%	27.1%	62.1%
	Percent age 15 or older with at least one dose, difference	15.0%	4.7%	10.4%	21.5%
	Percent age 65 or older	10.5%	5.7%	5.6%	16.6%
	Percent employed in health care or social assistance	11.2%	2.4%	8.3%	14.2%
	Percent under poverty line	17.1%	9.0%	5.7%	30.1%
	Percent with Medicaid or other means-tested public health insurance	8.0%	6.1%	1.8%	16.3%
	Percent without health insurance	24.3%	11.9%	8.2%	39.2%
	Percent without internet access	18.3%	12.7%	5.5%	39.9%
	Percent Black	22.9%	19.5%	5.8%	55.4%
	Percent Hispanic	36.5%	20.7%	13.9%	65.1%
	Percent Asian	5.2%	9.7%	0.2%	8.3%
Overall ($n = 552$)	Percent age 15 or older with at least one dose, March	28.0%	9.0%	17.9%	39.4%
	Percent age 15 or older with at least one dose, April	42.5%	12.4%	28.1%	59.2%
	Percent age 15 or older with at least one dose, difference	14.5%	4.6%	9.7%	21.4%
	Percent age 65 or older	12.8%	4.8%	7.2%	18.9%
	Percent employed in health care or social assistance	14.6%	5.4%	8.9%	22.7%
	Percent under poverty line	17.1%	9.6%	6.1%	31.4%
	Percent with Medicaid or other means-tested public health insurance	15.3%	11.1%	3.1%	31.0%
	Percent without health insurance	14.3%	10.6%	4.0%	31.4%
	Percent without internet access	15.3%	9.3%	4.2%	27.1%
	Percent Black	19.9%	23.3%	1.9%	57.7%
	Percent Hispanic	32.7%	23.9%	6.8%	69.0%
	Percent Asian	9.2%	10.8%	0.7%	23.3%

was to compound the effects of spatial scale that we discuss in Section e3.1: underestimating associations and disparities.

Table e3.5 provides a partial assessment of the level of measurement error stemming from spatial interpolation. The table compares the official ACS population estimates with the sums of the interpolated populations of the target zones. For all cities except Houston and for the eight cities combined, the interpolated population was within one percent of the official estimate. The much greater difference between the estimates for Houston is probably attributable to its irregular physical boundaries, as illustrated in Figure e3.1. This layout would be challenging for any method of spatial interpolation. On average, however, Table e3.5 suggests our overlay interpolation procedure distributed populations with considerable accuracy. This comparison is insufficient to rule out information bias from the interpolation, but it suggests the biasing effects of resulting measurement error were minor overall.

e4 Analytical approach

e4.1 Model specification and estimation

We estimated standard linear models (SLMs) by weighted least squares (LS) and spatial error models (SEMs) with nearest-neighbor spatial weights by maximum likelihood.[63–67] Formally, for observations of $n = 552$ units on ρ independent variables and $k = 8$ nearest neighbors, the SLM was

$$Qy = Q\alpha + QX\beta + Q\varepsilon, \tag{e4.1}$$

and the SEM was

$$\begin{aligned} Qy &= Q\alpha + QX\beta + Qu \\ &= Q\alpha + QX\beta + Q(\lambda W u + \varepsilon) \\ &= Q\alpha + QX\beta + Q\left(I_n - \lambda W\right)^{-1} \varepsilon, \end{aligned} \tag{e4.2}$$

with

$$Q = \begin{pmatrix} q_1^{15+} & 0 & \cdots & 0 \\ 0 & q_2^{15+} & \cdots & 0 \\ \vdots & \vdots & \ddots & \vdots \\ 0 & 0 & \cdots & q_n^{15+} \end{pmatrix}^{1/2}; \tag{e4.3}$$

$$W = \begin{pmatrix} \sum_{j=1}^{n} g_{1j} & 0 & \cdots & 0 \\ 0 & \sum_{j=1}^{n} g_{2j} & \cdots & 0 \\ \vdots & \vdots & \ddots & \vdots \\ 0 & 0 & \cdots & \sum_{j=1}^{n} g_{nj} \end{pmatrix}^{-1} G = \frac{G}{k}; \tag{e4.4}$$

$$G = \begin{pmatrix} g_{11} & g_{12} & \cdots & g_{1n} \\ g_{21} & g_{22} & \cdots & g_{2n} \\ \vdots & \vdots & \ddots & \vdots \\ g_{n1} & \cdots g_{n2} & \cdots & g_{nn} \end{pmatrix}; \tag{e4.5}$$

$$g_{ij} = \begin{cases} 1 & j \in h_i \\ 0 & j \notin h_i \end{cases}; \tag{e4.6}$$

where y was an $n \times 1$ vector of observations on the outcome variable; X was an $n \times \rho$ matrix of observations on the independent variables; I_n was an identity matrix of size n; q_i^{15+} was the estimated population age 15 and older of unit i; h_i indexed the k units closest to unit i; α was the intercept (constant) parameter to be estimated; β was a $1 \times \rho$ vector of coefficients to be estimated; ε was an $n \times 1$ vector of disturbances; λ was a scalar parameter to be estimated measuring average spatial autocorrelation in ε, conditional on W; and population weights matrix Q, spatial weights matrix W, and spatial links matrix G were square, of order n.

We report expanded step-wise results of both sets of models in Tables e4.1 and e4.2, and we compare the estimates between the SEMs and SLMs that included all independent variables in Figure e4.1. For the March and April outcomes, the coefficient estimates differed significantly or very nearly significantly between the models for three of the SES indicators. The magnitudes of the coefficients for the insurance-related variables decreased from the SLM to the SEM but remained negative, while the coefficient for the poverty variable changed from slightly positive to slightly negative.

e4.2 Spatial heterogeneity and modeling strategy

Testing for and modeling spatial effects

Estimating SLMs by LS, the most common tool for analyzing high-dimensional relationships among variables, is unbiased and efficient in many settings.[64] In the presence of spatial effects, however, LS estimates are inaccurate and/or require larger samples to model relationships.[65,66,68,69] There are two basic classes of spatial effects: spatial heterogeneity and spatial dependence.

Spatial heterogeneity "is related to the lack of stability over space of the ... relationships under study. More precisely, this implies that functional forms and parameters vary with location and are not homogeneous throughout the data set."[65p9] It was plausible that the relationships among vaccination, priority population composition, socioeconomic composition, and racial/ethnic composition varied from unit to unit but were spatially clustered, even within cities. In principle, the likely sources of spatial heterogeneity could be modeled as independent variables; in practice, they were unobserved. Potential unmeasurable sources of spatial heterogeneity included past levels of exposure to COVID-19, hyperlocal idiosyncrasies in the effects or implementation of vaccination policies, and cultural influences.

Spatial dependence, which includes spillover effects or externalities, is "the existence of a functional relationship between what happens at one point in space and what happens elsewhere."[65p11] Spatial dependence seemed less likely to contaminate this analysis, but it

Table e4.1: Step-wise standard linear model (SLM) estimates of COVID-19 vaccination levels in the population age 15 and older of ZIP Codes across eight large U.S. cities, March and April 2021

	% vaccinated, March				% vaccinated, April				Difference			
% 65+	1.068***	0.670***	0.606***	0.593***	1.132***	0.563*	0.442**	0.422***	0.064	−0.107	−0.164	−0.171*
	(0.171)	(0.122)	(0.080)	(0.044)	(0.247)	(0.229)	(0.139)	(0.104)	(0.081)	(0.111)	(0.087)	(0.086)
% health care workers	−0.531**	−0.399***	0.035	−0.050	−1.060***	−0.900***	−0.267	−0.408	−0.530***	−0.501***	−0.302*	−0.358***
	(0.173)	(0.116)	(0.337)	(0.215)	(0.248)	(0.164)	(0.470)	(0.277)	(0.079)	(0.056)	(0.141)	(0.083)
% under poverty line		0.019		0.123***		0.018		0.192*		−0.000		0.069
		(0.057)		(0.023)		(0.083)		(0.076)		(0.037)		(0.055)
% w/ Medicaid, etc.		−0.114		−0.276***		−0.130		−0.400***		−0.016		−0.124**
		(0.073)		(0.024)		(0.111)		(0.048)		(0.046)		(0.038)
% w/o health insurance		−0.376**		−0.569***		−0.574***		−0.885***		−0.198***		−0.316***
		(0.116)		(0.049)		(0.167)		(0.072)		(0.055)		(0.025)
% w/o internet access		−0.169		−0.036		−0.237		−0.032		−0.068		0.004
		(0.112)		(0.081)		(0.169)		(0.121)		(0.061)		(0.041)
% Black			−0.194**	−0.077			−0.257*	−0.085			−0.063	−0.009
			(0.069)	(0.048)			(0.104)	(0.070)			(0.039)	(0.030)
% Hispanic			−0.156**	0.073**			−0.223**	0.119**			−0.067**	0.046**
			(0.056)	(0.028)			(0.075)	(0.036)			(0.025)	(0.018)
% Asian			−0.061***	0.111***			−0.005	0.253***			0.056***	0.142***
			(0.010)	(0.026)			(0.020)	(0.048)			(0.016)	(0.026)
Chicago†	0.023*	0.023***	0.045**	0.040***	0.024	0.026**	0.058*	0.053***	0.001	0.003	0.013*	0.012**
	(0.010)	(0.006)	(0.017)	(0.011)	(0.015)	(0.009)	(0.024)	(0.015)	(0.005)	(0.004)	(0.007)	(0.005)
Houston†	0.020	0.062**	0.059*	0.085***	−0.026	0.044	0.035	0.079*	−0.045***	−0.018	−0.025*	−0.006
	(0.018)	(0.021)	(0.029)	(0.022)	(0.026)	(0.033)	(0.040)	(0.032)	(0.008)	(0.012)	(0.011)	(0.011)
Phoenix†	−0.007	0.001	−0.005	0.013	−0.069**	−0.053**	−0.056**	−0.027	−0.062***	−0.054***	−0.051***	−0.040***
	(0.016)	(0.012)	(0.015)	(0.011)	(0.023)	(0.019)	(0.020)	(0.015)	(0.007)	(0.007)	(0.006)	(0.006)
Philadelphia†	−0.021***	−0.024***	−0.032***	−0.017***	−0.031***	−0.034***	−0.045***	−0.022***	−0.010***	−0.010*	−0.013***	−0.005
	(0.004)	(0.007)	(0.006)	(0.005)	(0.005)	(0.010)	(0.008)	(0.006)	(0.002)	(0.004)	(0.002)	(0.004)
San Antonio†	0.008	0.026*	0.039	0.008	−0.037*	−0.003	0.019	−0.023	−0.045***	−0.029**	−0.021**	−0.031***
	(0.011)	(0.013)	(0.022)	(0.012)	(0.016)	(0.022)	(0.029)	(0.018)	(0.005)	(0.009)	(0.007)	(0.006)
San Diego†	0.046***	0.021**	0.040***	0.021*	0.030	−0.003	0.022*	−0.005	−0.016**	−0.025***	−0.019***	−0.026***
	(0.012)	(0.007)	(0.007)	(0.008)	(0.017)	(0.013)	(0.009)	(0.012)	(0.005)	(0.007)	(0.003)	(0.005)
Dallas†	−0.002	0.047*	0.033	0.071***	−0.043	0.037	0.013	0.077**	−0.041***	−0.010	−0.020	0.006
	(0.018)	(0.020)	(0.028)	(0.019)	(0.026)	(0.030)	(0.038)	(0.027)	(0.008)	(0.010)	(0.010)	(0.009)
Intercept	0.208***	0.327***	0.269***	0.273***	0.442***	0.614***	0.520***	0.526***	0.234***	0.287***	0.251***	0.252***
	(0.053)	(0.019)	(0.023)	(0.023)	(0.075)	(0.038)	(0.032)	(0.028)	(0.023)	(0.021)	(0.016)	(0.015)
Adjusted R^2	0.369	0.619	0.557	0.677	0.363	0.630	0.565	0.704	0.420	0.594	0.575	0.681
Residual Moran's I	0.344***	0.264***	0.284***	0.250***	0.350***	0.241***	0.282***	0.222***	0.340***	0.235***	0.274***	0.202***
Spatial heterogeneity	302.737***	173.438***	205.156***	156.517***	314.961***	145.301***	203.595***	123.383***	296.913***	138.244***	191.166***	101.537***

SLMs estimated by weighted LS (see Equation e4.1). $N = 552$ ZIP Code-based units across eight of the 10 most populous U.S. cities.
† fixed effects (reference: New York). All models weighted by estimated population age 15 and older. Percentages scaled from zero to one.
Heteroskedasticity-robust standard errors clustered by state in parentheses. $^{***}p < 0.001$; $^{**}p < 0.01$; $^{*}p < 0.05$.
Moran's I p-values calculated by permutation bootstrap (9,999 iterations). Spatial heterogeneity test statistic from simple Lagrange multiplier test for error dependence.
"Health care workers" refers to individuals employed in health care and social assistance. "Medicaid, etc." refers to Medicaid or any other means-tested public health insurance.
The "% vaccinated" is the percent of the population age 15 and older with at least one dose of a COVID-19 vaccine.

Table e4.2: Step-wise spatial error model (SEM) estimates of COVID-19 vaccination levels in the population age 15 and older of interpolated units of analysis across eight large U.S. cities, March and April 2021

	% vaccinated, March				% vaccinated, April				Difference			
% 65+	1.031***	0.669***	0.671***	0.593***	1.077***	0.581***	0.593***	0.470***	0.045	−0.090	−0.078	−0.122*
	(0.161)	(0.063)	(0.028)	(0.048)	(0.226)	(0.113)	(0.072)	(0.075)	(0.071)	(0.062)	(0.053)	(0.054)
% health care workers	−0.270	−0.238	0.304	0.147	−0.542	−0.560**	0.202	−0.063	−0.269***	−0.309***	−0.100	−0.201***
	(0.252)	(0.157)	(0.378)	(0.257)	(0.326)	(0.190)	(0.490)	(0.309)	(0.074)	(0.034)	(0.113)	(0.055)
% under poverty line		−0.189**		−0.102*		−0.284**		−0.138**		−0.097*		−0.039
		(0.066)		(0.043)		(0.093)		(0.051)		(0.038)		(0.023)
% w/ Medicaid, etc.		0.017		−0.102***		0.095		−0.127**		0.082		−0.021
		(0.098)		(0.024)		(0.146)		(0.046)		(0.054)		(0.029)
% w/o health insurance		−0.318***		−0.418***		−0.482***		−0.655***		−0.161***		−0.234***
		(0.088)		(0.039)		(0.119)		(0.053)		(0.042)		(0.023)
% w/o internet access		−0.132*		−0.040		−0.177		−0.036		−0.045		0.003
		(0.067)		(0.051)		(0.091)		(0.060)		(0.026)		(0.011)
% Black			−0.236**	−0.111			−0.311*	−0.132			−0.074	−0.021
			(0.090)	(0.061)			(0.129)	(0.084)			(0.041)	(0.025)
% Hispanic			−0.149*	0.041			−0.199*	0.076			−0.049*	0.036***
			(0.059)	(0.035)			(0.079)	(0.041)			(0.025)	(0.010)
% Asian			−0.021	0.101			0.056	0.230*			0.076*	0.127***
			(0.053)	(0.067)			(0.085)	(0.103)			(0.033)	(0.037)
Chicago†	0.054***	0.058***	0.089***	0.078***	0.066**	0.078***	0.119***	0.103***	0.009	0.018***	0.028***	0.026***
	(0.015)	(0.007)	(0.023)	(0.015)	(0.020)	(0.011)	(0.029)	(0.019)	(0.005)	(0.004)	(0.006)	(0.004)
Houston†	0.053*	0.092***	0.092**	0.113***	0.031	0.099**	0.086*	0.124***	−0.023**	0.008	−0.007	0.012
	(0.025)	(0.022)	(0.032)	(0.025)	(0.033)	(0.031)	(0.041)	(0.034)	(0.008)	(0.010)	(0.009)	(0.009)
Phoenix†	−0.011	−0.011	−0.003	0.002	−0.077*	−0.068***	−0.055*	−0.041*	−0.066***	−0.060***	−0.052***	−0.045***
	(0.025)	(0.014)	(0.020)	(0.016)	(0.034)	(0.020)	(0.026)	(0.020)	(0.009)	(0.007)	(0.007)	(0.005)
Philadelphia†	−0.052***	−0.027***	−0.037***	−0.009	−0.067***	−0.029***	−0.040***	−0.001	−0.011***	−0.000	−0.001	0.009**
	(0.003)	(0.006)	(0.006)	(0.006)	(0.004)	(0.009)	(0.009)	(0.009)	(0.001)	(0.004)	(0.003)	(0.003)
San Antonio†	0.001	0.024	0.036	0.016	−0.036	0.007	0.021	−0.004	−0.035***	−0.016	−0.015	−0.020***
	(0.022)	(0.016)	(0.029)	(0.020)	(0.031)	(0.025)	(0.039)	(0.025)	(0.008)	(0.009)	(0.009)	(0.006)
San Diego†	0.039*	0.019*	0.040***	0.015	0.038	0.011	0.037*	0.001	0.004	−0.005	−0.002	−0.014***
	(0.016)	(0.007)	(0.012)	(0.011)	(0.021)	(0.011)	(0.015)	(0.014)	(0.005)	(0.004)	(0.004)	(0.003)
Dallas†	−0.004	0.075**	0.058	0.097**	−0.053	0.082*	0.041	0.110**	−0.051***	0.006	−0.018	0.012
	(0.031)	(0.024)	(0.044)	(0.029)	(0.044)	(0.034)	(0.060)	(0.041)	(0.012)	(0.010)	(0.015)	(0.012)
Intercept	0.155*	0.286***	0.201***	0.232***	0.339***	0.522***	0.393***	0.443***	0.184***	0.232***	0.192***	0.208***
	(0.070)	(0.017)	(0.034)	(0.030)	(0.094)	(0.031)	(0.040)	(0.034)	(0.024)	(0.020)	(0.014)	(0.014)
Lambda	0.717	0.670	0.705	0.675	0.742	0.690	0.721	0.678	0.777	0.750	0.743	0.710
AIC	−1,468.592	−1,675.858	−1,640.426	−1,759.093	−1,117.134	−1,323.141	−1,295.013	−1,430.062	−2,263.635	−2,378.652	−2,391.239	−2,481.893
Residual Moran's I	0.029	0.034*	−0.008	0.027	0.021	0.018	−0.013	0.014	0.006	−0.011	−0.012	−0.015

SEMs estimated by maximum likelihood with row-standardized nearest-neighbor spatial weighting, $k = 8$ (see Equation e4.2). $N = 552$ ZIP Code-based units across eight of the 10 most populous U.S. cities.
† fixed effects (reference: New York). All models weighted by estimated population age 15 and older. Percentages scaled from zero to one.
Heteroskedasticity-robust standard errors clustered by state in parentheses. $^{***}p < 0.001$; $^{**}p < 0.01$; $^{*}p < 0.05$.
Lambda: estimated autocorrelation parameter; see Equation e4.2. AIC: Akaike information criterion. Moran's I p-values calculated by permutation bootstrap (9,999 iterations).
"Health care workers" refers to individuals employed in health care and social assistance. "Medicaid, etc." refers to Medicaid or any other means-tested public health insurance.
The "% vaccinated" is the percent of the population age 15 and older with at least one dose of a COVID-19 vaccine.

Figure e4.1: Estimated coefficients from standard linear model (SLM) and spatial error model (SEM) estimates of COVID-19 vaccination levels in the population age 15 and older of interpolated units of analysis across eight large U.S. cities, March and April 2021

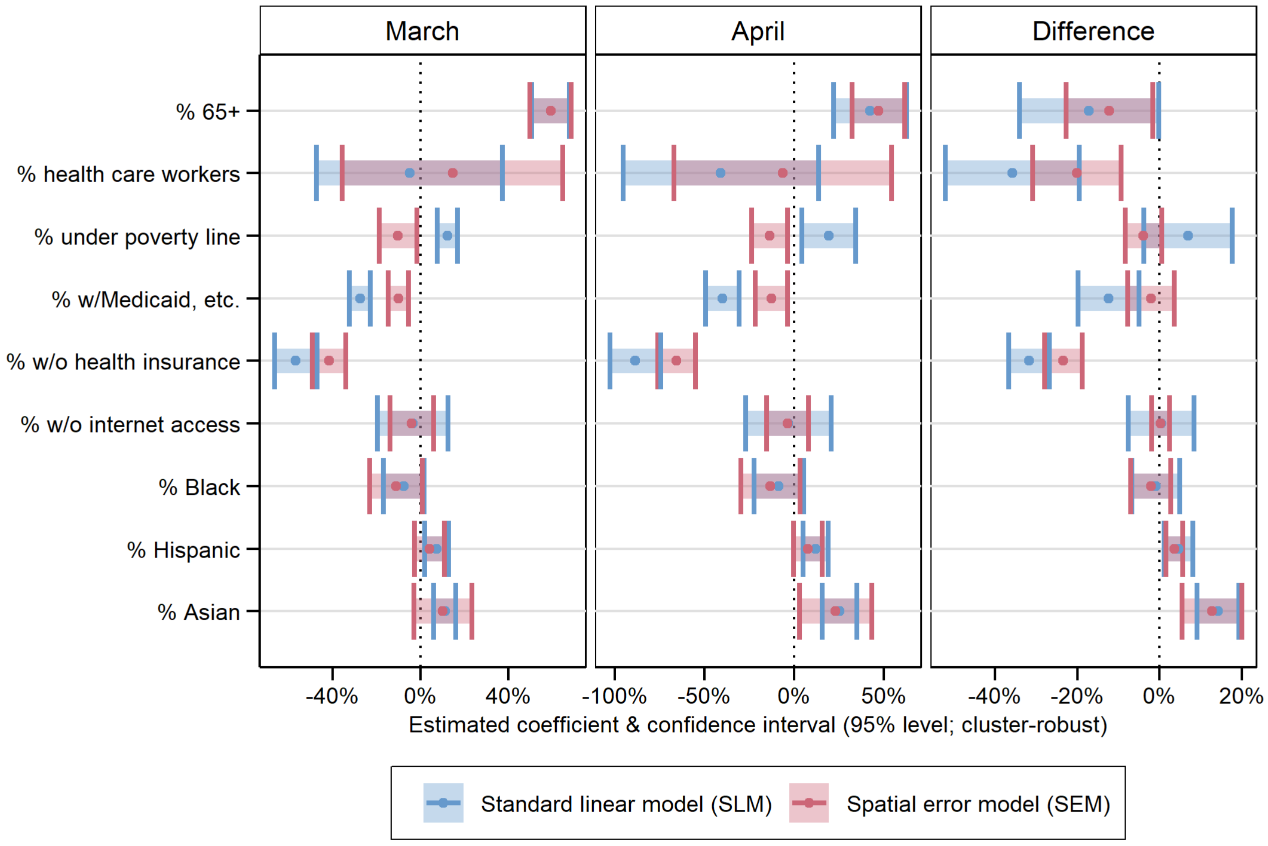

"Health care workers" refers to individuals employed in health care and social assistance.
"Medicaid, etc." refers to Medicaid or any other means-tested public health insurance.
The "% vaccinated" is the percent of the population age 15 and older with at least one dose of a COVID-19 vaccine.

was not implausible. For example, zero-sum dynamics could have emerged between neighboring units when vaccine doses were scarce. Similarly, peer-effects might have caused uneven diffusion of vaccination across space.

Testing for and modeling spatial effects requires the researcher to specify the structure of spatial relationships. This structure is conventionally represented in matrix form as in Equation e4.4.[70–72] We evaluated various common spatial weight matrix specifications, including adjacency ("queen" and "rook" contiguity), inverse distance, power distance, exponential distance, and nearest-neighbor weighting. We defined distance between units as the number of kilometers between their geometric centers, or centroids.[57,73]

We ultimately used a row-standardized k nearest-neighbors scheme, with $k = 8$. With this specification of W, we assumed relationships were best measured relative to the eight other units closest to each unit. Each unit's eight nearest-neighbors contributed equally to the spatial influence on it, and each unit's spatial weights summed to one; each nearest-neighbor contributed one-eighth of the spatial influence on unit i.

In our multi-city analysis, nearest-neighbor weighting produced more consistent weights than contiguity- or distance-based weighting. The units of analysis were irregularly sized and sometimes physically discontinuous within cities, and density and sprawl varied considerably across cities (see Figure e3.1 and Table e3.4). Nearest-neighbor weighting ensured each unit was weighted relative to the same number of other units. It also avoided fluctuations from the varying spatial distributions of cities' populations and the arbitrary boundaries of ZCTAs. Compared to contiguity-based weights, the nearest-neighbor approach was particularly advantageous for units on the edges of cities' boundaries and units with few or zero adjacent neighbors (such as islands). Compared to distance-based weights, it avoided skew that could result from atypically large units that received artificially low weights due to their exaggerated inter-centroid distances with other units. Overall, nearest-neighbor weights with $k = 8$ struck an effective balance between the more rigid assumptions of contiguity-based weights and the perhaps overly-encompassing assumptions of distance-based weights.

We first tested for spatial effects by evaluating clustering in the SLM residuals using Moran's I.[74] A spatial complement to the conventional correlation coefficient, I measures spatial autocorrelation on a scale from negative one to one. To determine whether I was statistically distinguishable from zero, we ran permutation tests with $9,999$ iterations.[73] We report Moran's I for the SLMs that included all independent variables in the first row of Table e4.3. For each model, I was positive and highly significant, indicating units closer to one another had more similar residuals than units farther from one another (positive spatial autocorrelation). The Moran's I test on the SLM residuals provided strong preliminary evidence of spatial effects, suggesting LS estimation was suboptimal in our setting.

Whereas I is a generic test statistic for spatial autocorrelation, Lagrange multiplier tests for SLM residuals help distinguish between types of spatial effects—i.e., to determine whether models exhibit spatial heterogeneity, dependence, or both.[75] We summarize the results of the Lagrange multiplier tests for the SLM residuals in the bottom four rows of Table e4.3.[73] The tests rejected the null hypothesis that the SLMs were free of spatial heterogeneity at $p < 0.001$. They retained the null hypothesis that the models were free of spatial dependence. These results held when testing for heterogeneity and dependence alone (simple tests) and when accounting for the simultaneous possibility of the other (robust tests). Lagrange multiplier tests strongly suggested the spatial relationships among our independent and dependent

Table e4.3: Tests for spatial effects in residuals of standard linear model (SLM) estimates of COVID-19 vaccination levels in the population age 15 and older of ZIP Codes across eight large U.S. cities, March and April 2021

Test	March	April	Difference
Moran's I	0.250^{***}	0.222^{***}	0.202^{***}
Spatial heterogeneity (simple)	156.517^{***}	123.383^{***}	101.537^{***}
Spatial dependence (simple)	2.879	1.990	2.001
Spatial heterogeneity (robust)	153.982^{***}	121.680^{***}	99.808^{***}
Spatial dependence (robust)	0.345	0.286	0.278

$^{***}p < 0.001$; $^{**}p < 0.01$; $^{*}p < 0.05$.
Moran's I p-values from permutation bootstrap (9,999 iterations).
Simple Lagrange multiplier tests evaluate one spatial effect.
Robust Lagrange multiplier tests allow for simultaneous effects.

variables were heterogeneous across space, but no evidence emerged for dependence among nearby units.

When spatial heterogeneity is present and spatial dependence absent, the SEM is a standard modeling approach.[65,66,72] SEMs account for unobserved, spatially clustered independent variables—spatial autoccorelation in the disturbances—that lead to spatial clustering in the associations among observed variables. LS estimation cannot capture these spatial dynamics. SEMs yield reliable estimates in the absence of spillover effects and reduce bias from confounding variables.[69] Another advantage of SEMs is interpretability. Unlike other spatial models, the independent variables are identical between SLMs and corresponding SEMs. The difference between SLMs and SEMs instead lies in the specification of the disturbances: SEMs introduce a spatially correlated vector of random effects, u in Equation e4.2. Interpreting SEM parameter estimates is thus comparable to interpreting SLM coefficients.[76] By estimating SEMs, we assumed that u was independent of X—i.e., that unmeasured spatially clustered variables were uncorrelated with the observed independent variables.

City fixed effects, population weights, and cluster-robust standard errors

There were three other key components to our modeling strategy. First, we included city fixed effects. Because state and local authorities oversaw vaccine distribution and local political contexts varied dramatically, potential sources of city-level heterogeneity abounded. The fixed effects adjusted for unobserved characteristics that units shared within cities.[77] They accounted for the possibility that average outcomes varied by city and purged associated confounding.

Second, we weighted the units of analysis by population using matrix Q. Population weighting addressed heteroskedasticity stemming from associations between the variance in vaccination outcomes and the number of residents of each unit.[78] The substantive assumption of unweighted models would have been that each unit represented an equal share of the process of vaccine distribution and uptake. Estimating sample-wide average associations among the independent and dependent variables required weighting by the population of each unit that could receive the vaccine.[79–81] Due to the weighting, more populous units

and cities influenced estimation more than less populous counterparts, consistent with their greater share of the sampled population. Table e3.5 contextualizes the relative influence of each city.

Third, we assessed statistical significance using heteroskedasticity-robust standard errors clustered by state (cluster-robust standard errors). While fixed effects netted out city-level unobserved heterogeneity, disturbances for units in the same state were vulnerable to clustering. (Of the eight cities, three were in Texas). We addressed this issue by computing standard errors using the product of the conventional "sandwich" estimator[82] and

$$\frac{\mu(n-1)}{(\mu-1)(n-\rho)}, \tag{e4.7}$$

where $\mu = 6$ was the number of clusters.[83,84] (This adjustment to the "sandwich" estimator is the default in many Stata commands). Calculated in this way, the standard errors were robust to within-state residual correlation.

e4.3 Simulations

Motivation

To illustrate relationships among the independent and dependent variables, we used a simulation approach. It was similar to conventional marginal effects analyses, which contextualize associations by visualizing the outcome that a model would predict given relevant values of the independent variables.[85,86] Because SEMs account for unobserved, spatially clustered variables, it is effectively impossible for researchers to identify appropriate points in the distribution at which to analyze trends and to predict the corresponding outcomes.[87,88] One approach would be to ignore the spatial structure of the disturbances and compute unit-level predictions for hypothetical observations, effectively treating the SEM as a SLM. This approach, however, distorts the modeled relationships.

Our simulations accounted for the spatial structure of the data. Instead of an estimate for individual hypothetical units as in marginal effects analyses, we derived sample-wide average estimated outcomes under instructive hypothetical conditions. We assumed every observation in the sample took on representative values at points of interest in the within-city distributions of the independent variables. We could thereby answer a simple counterfactual question: if every unit sat at the same place in its city's population distribution and were subject to the trends suggested by the SEM estimation, how would average vaccination outcomes compare to the observed averages? Figure e4.2 illustrates expanded results of the simulations.

As we indicate in Section e2.2, this simulation approach contextualized disparities more comprehensively than interpreting coefficients alone. Table e4.2 and Figure e4.1 show that the outcomes' estimated associations with percents Black and Hispanic were statistically insignificant in March and April; average vaccination outcomes did not change systematically with Black and Hispanic populations, conditional on vaccine priority populations, socioeconomic composition, and the spatial relationships specified in W. In isolation, these results suggest that economimc—not racial/ethnic—inequality explained variation in vaccination outcomes. By accounting for unequal distributions of SES and vaccine priority pop-

Figure e4.2: Simulated COVID-19 vaccination levels by racial/ethnic and socioeconomic composition in the population age 15 and older of ZIP Codes across eight large U.S. cities, March and April 2021 (expanded)

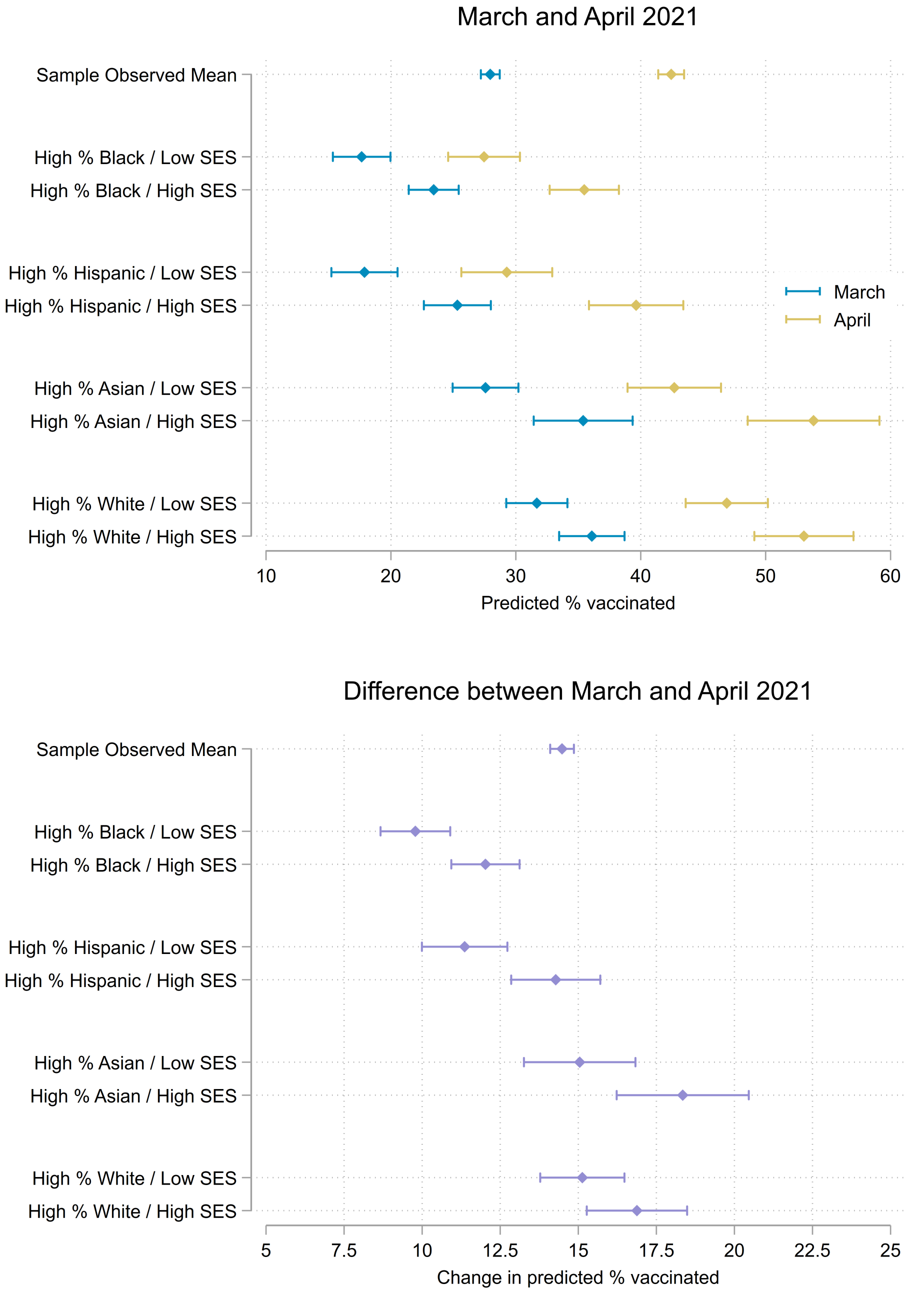

Dots represent simulated sample-wide means assuming each ZIP Code had a given socioeconomic and racial/ethnic composition. Bars represent bootstrapped confidence intervals (95% level). We defined low and high levels as below the 10th and above the 90th within-city percentiles, respectively. We defined SES levels by setting all four SES variables to the same within-city percentiles within each scenario. We set other independent variables to within-city averages in each scenario. We include the true (observed) sample-wide average values of the dependent variable on the top row for comparison. The “% vaccinated” is the percent of the population age 15 and older with at least one dose of a COVID-19 vaccine.

Table e4.4: Plausible values for independent variables used in simulations of COVID-19 vaccination levels by racial/ethnic and socioeconomic composition in the population age 15 and older of ZIP Codes across eight large U.S. cities, March and April 2021

Racial/ ethnic group	Group popula- tion	SES	City	Age 65+	Health care workers	Under poverty line	w/ Medi- caid, etc.	w/o health insurance	w/o internet access	Black	Hispanic	Asian
Asian	low	low	New York	13.2%	24.3%	35.7%	45.5%	12.5%	24.5%	47.0%	40.9%	1.9%
			Chicago	15.5%	19.1%	38.6%	38.3%	14.9%	35.0%	80.3%	14.3%	0.2%
			Houston	11.3%	11.2%	34.3%	19.6%	42.6%	41.7%	34.1%	59.4%	0.6%
			Phoenix	7.7%	10.5%	32.7%	27.7%	26.8%	23.5%	6.9%	66.0%	0.8%
			Philadelphia	12.3%	24.4%	45.7%	49.6%	13.8%	39.7%	65.9%	26.6%	1.1%
			San Antonio	12.3%	13.3%	34.3%	17.8%	33.0%	36.1%	3.5%	89.0%	0.3%
			San Diego	11.4%	11.4%	11.1%	9.9%	9.4%	5.6%	3.4%	18.4%	4.4%
			Dallas	10.8%	11.8%	32.0%	22.7%	39.2%	45.7%	39.5%	53.9%	0.2%
		high	New York	13.2%	24.3%	8.1%	16.6%	6.1%	13.9%	47.0%	40.9%	1.9%
			Chicago	15.5%	19.1%	24.1%	25.8%	10.2%	21.2%	80.3%	14.3%	0.2%
			Houston	11.3%	11.2%	19.6%	9.7%	21.8%	13.3%	34.1%	59.4%	0.6%
			Phoenix	7.7%	10.5%	13.6%	11.6%	13.0%	8.7%	6.9%	66.0%	0.8%
			Philadelphia	12.3%	24.4%	25.9%	23.6%	8.3%	23.4%	65.9%	26.6%	1.1%
			San Antonio	12.3%	13.3%	24.8%	13.3%	25.8%	22.5%	3.5%	89.0%	0.3%
			San Diego	11.4%	11.4%	9.0%	6.9%	7.3%	3.3%	3.4%	18.4%	4.4%
			Dallas	10.8%	11.8%	23.5%	9.9%	25.8%	35.2%	39.5%	53.9%	0.2%
	high	low	New York	16.7%	16.4%	22.6%	37.2%	12.9%	20.3%	5.7%	22.8%	36.6%
			Chicago	13.6%	13.8%	20.1%	13.8%	11.1%	10.4%	9.8%	9.8%	19.2%
			Houston	11.6%	11.0%	22.1%	11.2%	37.9%	18.6%	17.5%	34.0%	15.0%
			Phoenix	11.0%	12.9%	11.0%	11.8%	9.8%	7.4%	6.7%	21.3%	7.2%
			Philadelphia	15.7%	20.3%	34.7%	16.5%	8.7%	19.0%	21.5%	6.3%	14.9%
			San Antonio	9.7%	12.8%	12.2%	3.1%	11.4%	4.1%	6.3%	42.4%	6.8%
			San Diego	10.7%	12.8%	20.0%	7.8%	6.1%	3.4%	1.7%	11.1%	30.6%
			Dallas	8.4%	11.0%	19.5%	8.1%	25.7%	15.3%	14.3%	24.4%	8.2%
		high	New York	16.7%	16.4%	6.8%	9.7%	4.3%	9.1%	5.7%	22.8%	36.6%
			Chicago	13.6%	13.8%	9.2%	1.0%	2.1%	2.3%	9.8%	9.8%	19.2%
			Houston	11.6%	11.0%	5.8%	1.6%	7.4%	2.5%	17.5%	34.0%	15.0%
			Phoenix	11.0%	12.9%	5.0%	3.7%	4.6%	2.8%	6.7%	21.3%	7.2%
			Philadelphia	15.7%	20.3%	12.2%	6.2%	3.6%	7.3%	21.5%	6.3%	14.9%
			San Antonio	9.7%	12.8%	3.2%	1.4%	5.3%	1.6%	6.3%	42.4%	6.8%
			San Diego	10.7%	12.8%	5.4%	4.2%	3.0%	1.6%	1.7%	11.1%	30.6%
			Dallas	8.4%	11.0%	7.3%	2.2%	11.5%	6.5%	14.3%	24.4%	8.2%
Black	low	low	New York	16.1%	15.3%	18.2%	33.5%	13.2%	19.1%	1.4%	18.6%	19.7%
			Chicago	12.1%	11.7%	15.6%	16.3%	19.7%	19.7%	1.8%	49.9%	6.6%
			Houston	12.2%	8.2%	24.4%	12.3%	40.4%	25.2%	3.9%	48.1%	5.4%
			Phoenix	12.7%	13.7%	11.9%	12.9%	12.5%	7.7%	2.1%	14.6%	7.5%
			Philadelphia	17.1%	15.5%	18.5%	16.6%	9.6%	12.9%	5.9%	10.3%	17.1%
			San Antonio	13.3%	12.9%	25.5%	14.2%	29.6%	28.2%	1.0%	78.8%	0.9%
			San Diego	12.5%	11.9%	9.1%	6.9%	7.9%	3.2%	1.3%	12.7%	11.4%
			Dallas	11.1%	11.0%	13.4%	5.6%	27.9%	13.2%	5.8%	31.7%	5.2%
		high	New York	16.1%	15.3%	5.0%	2.3%	2.1%	2.2%	1.4%	18.6%	19.7%
			Chicago	12.1%	11.7%	8.9%	9.5%	11.1%	12.9%	1.8%	49.9%	6.6%
			Houston	12.2%	8.2%	5.2%	1.5%	7.4%	2.5%	3.9%	48.1%	5.4%
			Phoenix	12.7%	13.7%	4.1%	3.4%	3.5%	1.6%	2.1%	14.6%	7.5%
			Philadelphia	17.1%	15.5%	8.4%	2.8%	2.3%	3.8%	5.9%	10.3%	17.1%
			San Antonio	13.3%	12.9%	5.4%	2.1%	7.8%	4.7%	1.0%	78.8%	0.9%
			San Diego	12.5%	11.9%	3.8%	4.7%	2.9%	1.8%	1.3%	12.7%	11.4%
			Dallas	11.1%	11.0%	3.6%	2.1%	8.9%	5.5%	5.8%	31.7%	5.2%

"Health care workers" refers to individuals employed in health care and social assistance.
"Medicaid, etc." refers to Medicaid or any other means-tested public health insurance.

Table e4.4: Plausible values for independent variables used in simulations of COVID-19 vaccination levels by racial/ethnic and socioeconomic composition in the population age 15 and older of ZIP Codes across eight large U.S. cities, March and April 2021

Racial/ ethnic group	Group popula- tion	SES	City	Age 65+	Health care workers	Under poverty line	w/ Medi- caid, etc.	w/o health insurance	w/o internet access	Black	Hispanic	Asian
Hispanic	high	low	New York	13.6%	24.3%	26.1%	34.4%	10.4%	21.1%	58.2%	21.8%	4.3%
			Chicago	14.2%	21.1%	38.3%	38.6%	13.8%	33.5%	90.9%	4.2%	0.4%
			Houston	11.4%	14.7%	27.0%	22.0%	31.5%	31.4%	52.4%	34.3%	3.0%
			Phoenix	8.7%	12.4%	31.3%	28.9%	24.2%	27.7%	12.6%	51.4%	2.7%
			Philadelphia	12.5%	27.4%	33.7%	33.3%	12.9%	28.9%	84.6%	3.3%	4.0%
			San Antonio	12.9%	14.5%	20.0%	11.3%	23.1%	19.2%	15.0%	51.8%	3.0%
			San Diego	9.3%	11.9%	26.8%	31.5%	20.2%	20.3%	11.4%	47.6%	13.2%
			Dallas	12.2%	13.2%	34.1%	22.6%	37.1%	45.9%	49.7%	32.2%	3.1%
		high	New York	13.6%	24.3%	11.5%	19.7%	6.5%	13.2%	58.2%	21.8%	4.3%
			Chicago	14.2%	21.1%	28.5%	30.4%	8.4%	22.5%	90.9%	4.2%	0.4%
			Houston	11.4%	14.7%	15.2%	8.3%	20.9%	12.0%	52.4%	34.3%	3.0%
			Phoenix	8.7%	12.4%	23.0%	19.7%	18.0%	19.1%	12.6%	51.4%	2.7%
			Philadelphia	12.5%	27.4%	23.1%	20.4%	8.9%	21.6%	84.6%	3.3%	4.0%
			San Antonio	12.9%	14.5%	5.0%	4.6%	13.3%	6.2%	15.0%	51.8%	3.0%
			San Diego	9.3%	11.9%	12.9%	18.0%	8.9%	9.7%	11.4%	47.6%	13.2%
			Dallas	12.2%	13.2%	14.5%	7.5%	25.9%	13.6%	49.7%	32.2%	3.1%
	low	low	New York	17.1%	19.3%	13.9%	21.3%	8.3%	16.8%	27.9%	8.0%	8.6%
			Chicago	15.0%	18.5%	38.9%	38.1%	13.2%	30.1%	84.0%	3.2%	1.3%
			Houston	13.7%	10.5%	18.1%	8.2%	19.9%	15.0%	13.7%	16.9%	10.5%
			Phoenix	13.7%	13.8%	6.7%	6.2%	7.7%	5.4%	2.2%	11.0%	7.4%
			Philadelphia	14.0%	22.6%	36.2%	36.4%	12.2%	37.7%	77.6%	3.0%	2.9%
			San Antonio	15.3%	14.3%	9.6%	3.2%	11.3%	11.7%	5.3%	34.5%	4.6%
			San Diego	14.8%	13.7%	10.1%	6.5%	4.0%	4.1%	1.2%	9.1%	23.5%
			Dallas	11.4%	11.0%	16.9%	5.3%	13.9%	8.6%	11.9%	14.7%	10.0%
		high	New York	17.1%	19.3%	3.6%	2.5%	1.7%	4.3%	27.9%	8.0%	8.6%
			Chicago	15.0%	18.5%	18.9%	20.9%	6.4%	18.2%	84.0%	3.2%	1.3%
			Houston	13.7%	10.5%	5.8%	1.6%	5.7%	4.2%	13.7%	16.9%	10.5%
			Phoenix	13.7%	13.8%	3.1%	2.9%	4.2%	1.8%	2.2%	11.0%	7.4%
			Philadelphia	14.0%	22.6%	16.4%	17.9%	7.0%	15.9%	77.6%	3.0%	2.9%
			San Antonio	15.3%	14.3%	3.8%	2.2%	7.0%	2.6%	5.3%	34.5%	4.6%
			San Diego	14.8%	13.7%	4.8%	3.9%	2.6%	1.6%	1.2%	9.1%	23.5%
			Dallas	11.4%	11.0%	6.7%	1.5%	6.7%	2.8%	11.9%	14.7%	10.0%
	high	low	New York	12.4%	20.3%	39.0%	47.5%	15.9%	27.1%	20.6%	60.2%	5.8%
			Chicago	11.2%	12.1%	28.8%	25.6%	24.2%	26.5%	17.8%	55.5%	5.7%
			Houston	8.9%	8.4%	36.6%	18.1%	43.0%	41.7%	11.4%	74.5%	2.8%
			Phoenix	5.9%	9.6%	32.5%	27.6%	28.6%	23.8%	5.8%	74.3%	2.1%
			Philadelphia	10.0%	20.5%	38.4%	37.9%	15.3%	30.5%	43.3%	31.7%	6.3%
			San Antonio	11.4%	13.2%	33.5%	17.2%	33.0%	35.4%	2.8%	89.6%	0.4%
			San Diego	10.7%	11.5%	25.5%	28.8%	20.9%	16.6%	8.4%	62.2%	12.1%
			Dallas	8.4%	10.0%	22.5%	12.2%	39.4%	29.2%	19.6%	64.8%	0.6%
		high	New York	12.4%	20.3%	14.7%	24.8%	8.8%	14.1%	20.6%	60.2%	5.8%
			Chicago	11.2%	12.1%	8.9%	12.8%	13.1%	16.8%	17.8%	55.5%	5.7%
			Houston	8.9%	8.4%	16.6%	7.8%	34.5%	13.6%	11.4%	74.5%	2.8%
			Phoenix	5.9%	9.6%	13.9%	15.5%	20.1%	11.4%	5.8%	74.3%	2.1%
			Philadelphia	10.0%	20.5%	23.2%	24.7%	11.7%	17.3%	43.3%	31.7%	6.3%
			San Antonio	11.4%	13.2%	20.7%	11.8%	25.0%	18.8%	2.8%	89.6%	0.4%
			San Diego	10.7%	11.5%	13.8%	23.0%	12.6%	12.0%	8.4%	62.2%	12.1%
			Dallas	8.4%	10.0%	16.8%	7.5%	27.5%	13.9%	19.6%	64.8%	0.6%

"Health care workers" refers to individuals employed in health care and social assistance.
"Medicaid, etc." refers to Medicaid or any other means-tested public health insurance.

Table e4.4: Plausible values for independent variables used in simulations of COVID-19 vaccination levels by racial/ethnic and socioeconomic composition in the population age 15 and older of ZIP Codes across eight large U.S. cities, March and April 2021

Racial/ ethnic group	Group popula- tion	SES	City	Age 65+	Health care workers	Under poverty line	w/ Medi- caid, etc.	w/o health insurance	w/o internet access	Black	Hispanic	Asian
White	low	low	New York	12.1%	24.3%	35.7%	47.1%	15.9%	25.5%	44.2%	43.9%	5.4%
			Chicago	13.9%	18.1%	37.4%	38.4%	22.0%	30.5%	76.6%	19.5%	0.2%
			Houston	10.7%	11.7%	36.0%	19.2%	47.3%	42.0%	35.2%	59.1%	1.2%
			Phoenix	6.5%	11.0%	27.6%	26.9%	27.0%	26.8%	9.9%	71.4%	1.6%
			Philadelphia	12.3%	25.9%	44.8%	48.6%	13.5%	39.7%	73.5%	16.0%	3.9%
			San Antonio	11.4%	13.2%	33.5%	17.2%	33.0%	35.4%	2.8%	89.6%	0.4%
			San Diego	10.9%	12.5%	23.8%	32.1%	20.2%	20.6%	8.0%	65.3%	12.1%
			Dallas	10.3%	10.4%	28.6%	16.5%	39.5%	43.3%	30.1%	62.8%	0.4%
		high	New York	12.1%	24.3%	9.9%	18.9%	6.6%	12.8%	44.2%	43.9%	5.4%
			Chicago	13.9%	18.1%	25.8%	25.4%	9.9%	23.9%	76.6%	19.5%	0.2%
			Houston	10.7%	11.7%	15.0%	8.1%	24.2%	11.4%	35.2%	59.1%	1.2%
			Phoenix	6.5%	11.0%	13.9%	15.5%	17.3%	11.4%	9.9%	71.4%	1.6%
			Philadelphia	12.3%	25.9%	29.7%	28.9%	9.4%	28.0%	73.5%	16.0%	3.9%
			San Antonio	11.4%	13.2%	20.7%	11.8%	25.0%	18.8%	2.8%	89.6%	0.4%
			San Diego	10.9%	12.5%	9.9%	18.5%	8.9%	10.0%	8.0%	65.3%	12.1%
			Dallas	10.3%	10.4%	22.5%	9.2%	25.2%	25.7%	30.1%	62.8%	0.4%
	high	low	New York	18.3%	13.6%	12.9%	20.4%	8.1%	15.6%	3.6%	11.4%	10.4%
			Chicago	14.0%	11.3%	9.4%	8.2%	8.9%	18.4%	3.1%	11.9%	7.9%
			Houston	12.9%	9.0%	9.9%	4.7%	11.7%	10.1%	6.1%	20.4%	10.2%
			Phoenix	14.6%	13.7%	9.4%	6.5%	7.5%	5.4%	1.9%	10.3%	8.1%
			Philadelphia	13.8%	16.9%	15.0%	15.9%	7.8%	11.1%	8.8%	7.5%	5.4%
			San Antonio	13.0%	13.6%	9.0%	3.3%	10.1%	5.0%	5.4%	36.4%	4.7%
			San Diego	16.8%	12.0%	11.1%	6.3%	8.0%	4.6%	1.9%	13.1%	7.8%
			Dallas	10.5%	11.2%	12.2%	4.5%	12.1%	8.0%	7.9%	17.4%	5.2%
		high	New York	18.3%	13.6%	3.9%	2.3%	2.2%	2.2%	3.6%	11.4%	10.4%
			Chicago	14.0%	11.3%	5.9%	3.5%	2.4%	3.9%	3.1%	11.9%	7.9%
			Houston	12.9%	9.0%	5.3%	1.5%	4.9%	3.6%	6.1%	20.4%	10.2%
			Phoenix	14.6%	13.7%	3.6%	3.2%	3.5%	1.6%	1.9%	10.3%	8.1%
			Philadelphia	13.8%	16.9%	8.4%	3.1%	3.1%	3.8%	8.8%	7.5%	5.4%
			San Antonio	13.0%	13.6%	3.7%	1.4%	4.8%	3.6%	5.4%	36.4%	4.7%
			San Diego	16.8%	12.0%	6.9%	4.1%	4.5%	3.4%	1.9%	13.1%	7.8%
			Dallas	10.5%	11.2%	4.4%	1.8%	7.4%	3.2%	7.9%	17.4%	5.2%

"Health care workers" refers to individuals employed in health care and social assistance.
"Medicaid, etc." refers to Medicaid or any other means-tested public health insurance.

ulations across racial/ethnic groups, however, the simulations revealed that the explanation is more complicated. Because economic inequality is racialized, areas with high Black and Hispanic populations lagged behind areas with high Asian and White populations—even though the coefficients for percents Black and Hispanic were insignificant and the latter was even slightly positive. Attending to direct and indirect channels of structural racism more accurately represented the inequality-generating process than focusing only on average conditional racial/ethnic disparities.

Implementation

We first identified plausible values of the independent variables, listed in Table e4.4. Within each city, we identified low and high levels (the 10th and 90th percentiles) of Black, Hispanic, Asian, and White populations. Given these racial/ethnic compositions, we set plausible low and high values of the four SES variables. We identified the within-city 10th and 90th percentiles of the SES variables among units with populations within five percentile points of the within-city low and high levels for the given racial/ethnic group (the fifth through 15th and 85th through 95th percentiles). We set all other independent variables to their weighted averages within the same ranges. For example, in the iteration identified in Figure e4.2 as "High % Black / Low SES," we assigned all units low values (10th percentile) for the SES variables and average values of other independent variables, among units with high Black populations (85th through 95th percentiles) within their respective cities.

Next, we simulated outcomes given the plausible values of the independent variables. For each combination of racial/ethnic and socioeconomic compositions, we set the variables for each unit in our data set to the corresponding within-city values in Table e4.4. (We assigned every unit within each city the same values for each independent variable in each iteration). For each unit, we then calculated the estimated values of the outcome under each scenario, using the parameter estimates from the full models in Table e4.2. We computed population-weighted means for the three outcomes under each hypothetical scenario.

Finally, we bootstrapped confidence intervals for the simulated sample means. We obtained 1,000 resamples of our original data set by sampling it with replacement.[89] We then adjusted the spatial weights matrix W accordingly for each resample. We assigned nearest neighbors according to h_i in the original sample, which maintained the spatial structure of the data across iterations. Next, we re-estimated the SEMs on each of the resamples. We simulated the outcome variables under each hypothetical population composition of interest by assigning each unit the corresponding value in Table e4.4 in the same manner as above. From the distribution of the 1,000 resulting population-weighted resample means for each outcome, we calculated pivot confidence intervals at the 95% level.[90] This bootstrap procedure yielded a non-parametric approximation of the uncertainty in the hypothetical outcomes.

e5 References

1. DiRago NV. *Replication Data for: "COVID-19 Vaccine Rollouts and the Reproduction of Urban Spatial Inequality: Disparities Within Large U.S. Cities in March and April 2021 by Racial/Ethnic and Socioeconomic Composition".* Harvard Dataverse; 2021. doi:10.7910/DVN/O6EVCZ.
2. Bates D, Chambers J, Dalgaard P, et al. *R: A Language and Environment for Statistical Computing.* Version 4.0.4. R Foundation for Statistical Computing; 2021. https://www.R-project.org/.
3. Wickham H, Averick M, Bryan J, et al. Welcome to the Tidyverse. *JOSS.* 2019;4(43):1686. doi:10.21105/joss.01686.
4. Wickham H. *ggplot2: Elegant Graphics for Data Analysis.* 2nd ed. Springer; 2016.
5. *Stata Statistical Software.* Version 16.1. StataCorp; 2020.
6. Montesano M, Slutsker J, Pezeshki G. *NYC Coronavirus Disease 2019 (COVID-19) Data.* New York City Department of Health and Mental Hygiene; 2021. https://github.com/nychealth/coronavirus-data/blob/68acaed9e700aca48294d20129d4ae0551a27561/README.md.
7. Montesano M. *ZCTA to MODZCTA.* New York City Department of Health and Mental Hygiene; 2020. https://github.com/nychealth/coronavirus-data/blob/68acaed9e700aca48294d20129d4ae0551a27561/Geography-resources/ZCTA-to-MODZCTA.csv.
8. Jacobsen LA, Mather M, Jarosz B, et al. *Understanding and Using American Community Survey Data: What All Data Users Need to Know.* U.S. Census Bureau; 2020. https://www.census.gov/programs-surveys/acs/guidance/handbooks/general.html.
9. Walker K, Herman M. *tidycensus: Load US Census Boundary and Attribute Data as "tidyverse" and "sf"-Ready Data Frames.* Version 0.11.4. Comprehensive R Archive Network; 2021. https://CRAN.R-project.org/package=tidycensus.
10. Weber M. *Economy and Society: An Outline of Interpretive Sociology.* University of California Press; 1978.
11. Giddens A. *The Class Structure of the Advanced Societies.* Hutchinson; 1973.
12. Breen R. Foundations of a neo-Weberian class analysis. In: Wright EO, ed. *Approaches to Class Analysis.* Cambridge University Press; 2005: 31–50.
13. *Alphabetical Indexes of Industries and Occupations.* U.S. Census Bureau; 2019. https://www.census.gov/topics/employment/industry-occupation/guidance/indexes.html.
14. *American Community Survey and Puerto Rico Community Survey: 2019 Subject Definitions.* U.S. Census Bureau; 2020. https://www.census.gov/programs-surveys/acs/technical-documentation/code-lists.2019.html.
15. Denton NA. Racial identity and census categories: can incorrect categories yield correct information? *Law Inequal.* 1997;15(1):83–97. https://scholarship.law.umn.edu/lawineq/vol15/iss1/4.

16. Anderson M, Fienberg SE. Race and ethnicity and the controversy over the US census. *Curr Sociol.* 2000;48(3):87–110. doi:10.1177/0011392100048003007.

17. Fasenfest D, Booza J, Metzger K. *Living Together: A New Look at Racial and Ethnic Integration in Metropolitan Neighborhoods, 1990–2000.* Brookings Institution; 2004. https://www.brookings.edu/research/living-together-a-new-look-at-racial-and-ethnic-integration-in-metropolitan-neighborhoods/.

18. Simon P, Piché V. Accounting for ethnic and racial diversity: the challenge of enumeration. *Ethn Racial Stud.* 2012;35(8):1357–1365. doi:10.1080/01419870.2011.634508.

19. Kayyali R. US Census classifications and Arab Americans: contestations and definitions of identity markers. *J Ethn Migr Stud.* 2013;39(8):1299–1318. doi:10.1080/1369183X.2013.778150.

20. Liebler CA, Porter SR, Fernandez LE, Noon JM, Ennis SR. America's churning races: race and ethnicity response changes between Census 2000 and the 2010 Census. *Demography.* 2017;54(1):259–284. doi:10.1007/s13524-016-0544-0.

21. Strmic-Pawl HV, Jackson BA, Garner S. Race counts: racial and ethnic data on the U.S. census and the implications for tracking inequality. *Sociol Race Ethn.* 2018;4(1):1–13. doi:10.1177/2332649217742869.

22. Cabrera JF, Dela Cruz RR. Spatially based rules for reducing multiple-race into single-race data. *City Community.* 2020;19(3):593–616. doi:10.1111/cico.12418.

23. *2019 TIGER/Line Shapefiles Technical Documentation.* U.S. Census Bureau; 2019. https://www.census.gov/programs-surveys/geography/technical-documentation/complete-technical-documentation/tiger-geo-line.2019.html.

24. Walker K. *tigris: Load Census TIGER/Line Shapefiles.* Version 1.0. Comprehensive R Archive Network; 2020. https://CRAN.R-project.org/package=tigris.

25. *Population & Housing Characteristics for Congressional Districts of the 103rd Congress.* U.S. Bureau of the Census; 2002. https://www.census.gov/library/publications/1992/dec/cph-4.html.

26. Krieger N, Waterman P, Chen JT, Soobader MJ, Subramanian SV, Carson R. ZIP Code caveat: bias due to spatiotemporal mismatches between ZIP Codes and US Census–defined geographic areas. *Am J Public Health.* 2002;92(7):1100–1102. doi:10.2105/AJPH.92.7.1100.

27. Grubesic TH, Matisziw TC. On the use of ZIP Codes and ZIP Code Tabulation Areas (ZCTAs) for the spatial analysis of epidemiological data. *Int J Health Geogr.* 2006;5(58). doi:10.1186/1476-072X-5-58.

28. Beyer KMM, Schultz AF, Rushton G. Using ZIP Codes as geocodes in cancer research. In: Rushton G, Armstrong MP, Gittler J, et al., eds. *Geocoding Health Data: The Use of Geographic Codes in Cancer Prevention and Control, Research, and Practice.* CRC; 2008: 37–68.

29. Grubesic TH. ZIP Codes and spatial analysis: problems and prospects. *Socioecon Plann Sci.* 2008;42(2):129–149. doi:10.1016/j.seps.2006.09.001.

30. Sampson RJ, Morenoff JD, Gannon-Rowley T. Assessing “neighborhood effects:” social processes and new directions in research. *Annu Rev Sociol.* 2002;28(1):443–478. doi:10.1146/annurev.soc.28.110601.141114.

31. Sharkey P, Faber JW. Where, when, why, and for whom do residential contexts matter?: moving away from the dichotomous understanding of neighborhood effects. *Annu Rev Sociol.* 2014;40(1):559–579. doi:10.1146/annurev-soc-071913-043350.

32. Jarmin RS. Census tracts for the 2020 Census—final criteria. *Fed Regist.* 2018;83(219):56277–56284. https://www.federalregister.gov/documents/2018/11/13/2018-24567/census-tracts-for-the-2020-census-final-criteria.

33. Coulton CJ, Korbin J, Chan T, Su M. Mapping residents’ perceptions of neighborhood boundaries: a methodological note. *Am J Community Psychol.* 2001;29(2):371–383. doi:10.1023/A:1010303419034.

34. Sastry N, Pebley AR, Zonta M. Neighborhood definitions and the spatial dimension of daily life in Los Angeles. *RAND Labor and Population Program Working Paper Series.* 2002;03–02. https://www.rand.org/pubs/drafts/DRU2400z8.html.

35. Weiss L, Ompad D, Galea S, Vlahov D. Defining neighborhood boundaries for urban health research. *Am J Prev Med.* 2007;32(6):S154–S159. doi:10.1016/j.amepre.2007.02.034.

36. Campbell E, Henly JR, Elliott DS, Irwin K. Subjective constructions of neighborhood boundaries: lessons from a qualitative study of four neighborhoods. *J Urban Aff.* 2009;31(4):461–490. doi:10.1111/j.1467-9906.2009.00450.x.

37. Coulton CJ, Jennings MZ, Chan T. How big is my neighborhood?: individual and contextual effects on perceptions of neighborhood scale. *Am J Community Psychol.* 2013;51(1):140–150. doi:10.1007/s10464-012-9550-6.

38. Jones M, Pebley AR. Redefining neighborhoods using common destinations: social characteristics of activity spaces and home census tracts compared. *Demography.* 2014;51(3):727–752. doi:10.1007/s13524-014-0283-z.

39. Sharp G, Denney JT, Kimbro RT. Multiple contexts of exposure: activity spaces, residential neighborhoods, and self-rated health. *Soc Sci Med.* 2015;146:204–213. doi:10.1016/j.socscimed.2015.10.040.

40. Wang Q, Phillips NE, Small ML, Sampson RJ. Urban mobility and neighborhood isolation in America’s 50 largest cities. *PNAS.* 2018;115(30):7735–7740. doi:10.1073/pnas.1802537115.

41. Browning CR, Calder CA, Boettner B, et al. Neighborhoods, activity spaces, and the span of adolescent exposures. *Am Sociol Rev.* 2021;86(2):201–233. doi:10.1177/0003122421994219.

42. Chaskin RJ. Perspectives on neighborhood and community: a review of the literature. *Soc Serv Rev.* 1997;71(4):521–547. doi:10.1086/604277.

43. Bates LK. Does neighborhood really matter?: comparing historically defined neighborhood boundaries with housing submarkets. *J Plan Educ Res.* 2006;26(1):5–17. doi:10.1177/0739456X05283254.

44. Clapp JM, Wang Y. Defining neighborhood boundaries: are census tracts obsolete? *J Urban Econ.* 2006;59(2):259–284. doi:10.1016/j.jue.2005.10.003.

45. Lee BA, Reardon SF, Firebaugh G, Farrell CR, Matthews SA, O'Sullivan D. Beyond the census tract: patterns and determinants of racial segregation at multiple geographic scales. *Am Sociol Rev.* 2008;73(5):766–791. doi:10.1177/000312240807300504.

46. Hart TC, Waller J. Neighborhood boundaries and structural determinants of social disorganization: examining the validity of commonly used measures. *West Crim Rev.* 2013;14(3):16–33.

47. Poorthuis A. How to draw a neighborhood?: the potential of big data, regionalization, and community detection for understanding the heterogeneous nature of urban neighborhoods. *Geographical Analysis.* 2018;50(2):182–203. doi:10.1111/gean.12143.

48. Krieger N, Chen JT, Waterman PD, Soobader MJ, Subramanian SV, Carson R. Geocoding and monitoring of US socioeconomic inequalities in mortality and cancer incidence: does the choice of area-based measure and geographic level matter? *Am J Epidemiol.* 2002;156(5):471–482. doi:10.1093/aje/kwf068.

49. Thomas AJ, Eberly LE, Davey Smith G, Neaton JD. ZIP-Code-based versus tract-based income measures as long-term risk-adjusted mortality predictors. *Am J Epidemiol.* 2006;164(6):586–590. doi:10.1093/aje/kwj234.

50. Schuurman N, Bell N, Dunn JR, Oliver L. Deprivation indices, population health and geography: an evaluation of the spatial effectiveness of indices at multiple scales. *J Urban Health.* 2007;84(4):591–603. doi:10.1007/s11524-007-9193-3.

51. Rey G, Jougla E, Fouillet A, Hémon D. Ecological association between a deprivation index and mortality in France over the period 1997 – 2001: variations with spatial scale, degree of urbanicity, age, gender and cause of death. *BMC Public Health.* 2009;9(1):33. doi:10.1186/1471-2458-9-33.

52. Parenteau MP, Sawada MC. The modifiable areal unit problem (MAUP) in the relationship between exposure to NO_2 and respiratory health. *Int J Health Geogr.* 2011;10(1):58. doi:10.1186/1476-072X-10-58.

53. Halonen JI, Vahtera J, Oksanen T, et al. Socioeconomic characteristics of residential areas and risk of death: is variation in spatial units for analysis a source of heterogeneity in observed associations? *BMJ Open.* 2013;3(4):e002474. doi:10.1136/bmjopen-2012-002474.

54. Lam NSN. Spatial interpolation methods: a review. *Am Cartogr.* 1983;10(2):129–150. doi:10.1559/152304083783914958.

55. Hawley K, Moellering H. A comparative analysis of areal interpolation methods. *Cartogr Geogr Inf Sci.* 2005;32(4):411–423. doi:10.1559/152304005775194818.

56. Pebesma E, Mailund T, Hiebert J. Measurement units in R. *R J.* 2016;8(2):486–494. doi:10.32614/RJ-2016-061.

57. Pebesma E. Simple features for R: standardized support for spatial vector data. *R J.* 2018;10(1):439–446. doi:10.32614/RJ-2018-009.

58. Rothman KJ, Greenland S, Lash TL. *Modern Epidemiology.* 3rd ed. Lippincott Williams & Wilkins; 2008.

59. Liu K. Measurement error and its impact on partial correlation and multiple linear regression analyses. *Am J Epidemiol.* 1988;127(4):864–874. doi:10.1093/oxfordjournals.aje.a114870.

60. Hutcheon JA, Chiolero A, Hanley JA. Random measurement error and regression dilution bias. *BMJ.* 2010;340:c2289. doi:10.1136/bmj.c2289.

61. Loken E, Gelman A. Measurement error and the replication crisis. *Science.* 2017;355(6325):584–585. doi:10.1126/science.aal3618.

62. Hausman J. Mismeasured variables in econometric analysis: problems from the right and problems from the left. *J Econ Perspect.* 2001;15(4):57–67. doi:10.1257/jep.15.4.57.

63. Ord K. Estimation methods for models of spatial interaction. *J Am Stat Assoc.* 1975;70(349):120–126. doi:10.1080/01621459.1975.10480272.

64. Seber GAF, Lee AJ. *Linear Regression Analysis.* 2nd ed. John Wiley & Sons; 2003.

65. Anselin L. *Spatial Econometrics: Methods and Models.* Springer; 1988.

66. LeSage JP, Pace RK. *Introduction to Spatial Econometrics.* Chapman & Hall/CRC; 2009.

67. Bivand R, Piras G. Comparing implementations of estimation methods for spatial econometrics. *J Stat Softw.* 2015;63(1):1–36. doi:10.18637/jss.v063.i18.

68. Pace RK, LeSage JP. Omitted variable biases of OLS and spatial lag models. In: Páez A, Le Gallo J, Buliung RN, Dall'erba S, eds. *Progress in Spatial Analysis: Methods and Applications.* Springer; 2010: 17–28.

69. Rüttenauer T. Spatial regression models: a systematic comparison of different model specifications using Monte Carlo experiments. *Sociol Methods Res.* 2019; doi:10.1177/0049124119882467.

70. Tiefelsdorf M, Griffith DA, Boots B. A variance-stabilizing coding scheme for spatial link matrices. *Environ Plan A.* 1999;31(1):165–180. doi:10.1068/a310165.

71. Dubin R. Spatial weights. In: Fotheringham AS, Rogerson P, eds. *The SAGE Handbook of Spatial Analysis.* SAGE; 2009: 125–157.

72. Smith TE. *Notebook on Spatial Data Analysis.* School of Engineering and Applied Science, University of Pennsylvania; 2014. https://www.seas.upenn.edu/~tesmith/NOTEBOOK/index.html.

73. Bivand RS, Wong DWS. Comparing implementations of global and local indicators of spatial association. *TEST.* 2018;27(3):716–748. doi:10.1007/s11749-018-0599-x.

74. Schabenberger O, Gotway CA. *Statistical Methods for Spatial Data Analysis.* Chapman & Hall/CRC; 2005.

75. Anselin L, Bera AK, Florax R, Yoon MJ. Simple diagnostic tests for spatial dependence. *Reg Sci Urban Econ.* 1996;26(1):77–104. doi:10.1016/0166-0462(95)02111-6.

76. Golgher AB, Voss PR. How to interpret the coefficients of spatial models: spillovers, direct and indirect effects. *Spat Demogr.* 2016;4(3):175–205. doi:10.1007/s40980-015-0016-y.

77. Gormley TA, Matsa DA. Common errors: how to (and not to) control for unobserved heterogeneity. *Rev Financ Stud.* 2014;27(2):617–661. doi:10.1093/rfs/hht047.

78. Solon G, Haider SJ, Wooldridge JM. What are we weighting for? *J Hum Resour.* 2015;50(2):301–316. doi:10.3368/jhr.50.2.301.

79. Kneip T, Bauer G. Did unilateral divorce laws raise divorce rates in Western Europe? *J Marriage Fam.* 2009;71(3):592–607. doi:10.1111/j.1741-3737.2009.00621.x.

80. Schnapp P. Identifying the effect of immigration on homicide rates in U.S. cities: an instrumental variables approach. *Homicide Stud.* 2015;19(2):103–122. doi:10.1177/1088767914528907.

81. Sharkey P, Torrats-Espinosa G, Takyar D. Community and the crime decline: the causal effect of local nonprofits on violent crime. *Am Sociol Rev.* 2017;82(6):1214–1240. doi:10.1177/0003122417736289.

82. Liang KY, Zeger SL. Longitudinal data analysis using generalized linear models. *Biometrika.* 1986;73(1):13–22. doi:10.1093/biomet/73.1.13.

83. Cameron AC, Miller DL. A practitioner's guide to cluster-robust inference. *J Hum Resour.* 2015;50(2):317–372. doi:10.3368/jhr.50.2.317.

84. Pustejovsky J. *clubSandwich: Cluster-Robust (Sandwich) Variance Estimators with Small-Sample Corrections.* Version 0.5.3. Comprehensive R Archive Network; 2021. https://CRAN.R-project.org/package=clubSandwich.

85. Brambor T, Clark WR, Golder M. Understanding interaction models: improving empirical analyses. *Polit Anal.* 2006;14(1):63–82. doi:10.1093/pan/mpi014.

86. Pepinsky TB. Visual heuristics for marginal effects plots. *Research & Politics.* 2018;5(1). doi:10.1177/2053168018756668.

87. Bivand R. Spatial econometrics functions in R: classes and methods. *J Geograph Syst.* 2002;4(4):405–421. doi:10.1007/s101090300096.

88. Goulard M, Laurent T, Thomas-Agnan C. About predictions in spatial autoregressive models: optimal and almost optimal strategies. *Spat Econ Anal.* 2017;12(2-3):304–325. doi:10.1080/17421772.2017.1300679.

89. Davison AC, Hinkley DV. *Bootstrap Methods and Their Application.* Cambridge University Press; 1997.

90. DiCiccio TJ, Efron B. Bootstrap confidence intervals. *Stat Sci.* 1996;11(3):189–228. doi:10.1214/ss/1032280214.
